# Integrating terrestrial laser scanning with functional–structural plant models to investigate ecological and evolutionary processes of forest communities

**DOI:** 10.1093/aob/mcab120

**Published:** 2021-10-05

**Authors:** Hannah O’Sullivan, Pasi Raumonen, Pekka Kaitaniemi, Jari Perttunen, Risto Sievänen

**Affiliations:** 1 Department of Life Sciences, Imperial College London, Silwood Park, Ascot, Berkshire, SL5 7PY, UK; 2 Royal Botanic Gardens, Kew, Richmond, UK; 3 Mathematics, Tampere University, Korkeakoulunkatu 7, FI-33720 Tampere, Finland; 4 Hyytiälä Forestry Field Station, Faculty of Agriculture and Forestry, University of Helsinki, Hyytiäläntie 124, FI-35500 Korkeakoski, Finland; 5 Natural Resources Institute Finland, Latokartanontie 9, 00790 Helsinki, Finland; 6 Långstrandintie 281, 10600 Tammisaari, Finland

**Keywords:** Functional–structural plant models, terrestrial laser scanning, plant architecture, forest dynamics, vegetation modelling, remote sensing

## Abstract

**Background:**

Woody plants (trees and shrubs) play an important role in terrestrial ecosystems, but their size and longevity make them difficult subjects for traditional experiments. In the last 20 years functional–structural plant models (FSPMs) have evolved: they consider the interplay between plant modular structure, the immediate environment and internal functioning. However, computational constraints and data deficiency have long been limiting factors in a broader application of FSPMs, particularly at the scale of forest communities. Recently, terrestrial laser scanning (TLS), has emerged as an invaluable tool for capturing the 3-D structure of forest communities, thus opening up exciting opportunities to explore and predict forest dynamics with FSPMs.

**Scope:**

The potential synergies between TLS-derived data and FSPMs have yet to be fully explored. Here, we summarize recent developments in FSPM and TLS research, with a specific focus on woody plants. We then evaluate the emerging opportunities for applying FSPMs in an ecological and evolutionary context, in light of TLS-derived data, with particular consideration of the challenges posed by scaling up from individual trees to whole forests. Finally, we propose guidelines for incorporating TLS data into the FSPM workflow to encourage overlap of practice amongst researchers.

**Conclusions:**

We conclude that TLS is a feasible tool to help shift FSPMs from an individual-level modelling technique to a community-level one. The ability to scan multiple trees, of multiple species, in a short amount of time, is paramount to gathering the detailed structural information required for parameterizing FSPMs for forest communities. Conventional techniques, such as repeated manual forest surveys, have their limitations in explaining the driving mechanisms behind observed patterns in 3-D forest structure and dynamics. Therefore, other techniques are valuable to explore how forests might respond to environmental change. A robust synthesis between TLS and FSPMs provides the opportunity to virtually explore the spatial and temporal dynamics of forest communities.

## INTRODUCTION

How individual trees and shrubs occupy 3-D space is a defining feature of forest ecosystem dynamics (Díaz and Cabido, 2001; McDowell *et al.*, 2020). Plant architecture, the physical form of a plant as derived from the balance between internal and external processes, is a central but often overlooked element of forest ecology and evolution (Barthélémy and Caraglio, 2007). Individual-level form and function, in combination with the environment, ultimately contributes to overall forest ecosystem level processes, determining the biodiversity found within them, as well as the regulation of energy, water and nutrient fluxes. There are still many unanswered questions regarding the mechanisms that give rise to observable patterns of biodiversity, form and function in forests worldwide (Givnish, 1999; Condit *et al.*, 2006; Bohn and Huth, 2017). Elucidating the role of structure in these mechanisms is a crucial step in predicting the ecological and evolutionary responses of vegetation to forecasted climate change, deforestation or species invasions at the scale of forests and also at the scale of individual trees and shrubs (henceforth referred to as ‘woody plants’).

One way to characterize the relationship between biodiversity and the structure of forest environments is through the lens of *structural diversity* (Tews *et al.*, 2004), which denotes ‘the physical arrangement and variability of the living and non-living biotic elements within forest stands’ (LaRue *et al.*, 2020), and acts on its own as a good predictor of many ecosystem functions (Felipe-Lucia *et al.*, 2018; LaRue *et al.*, 2019). Recently, efforts have been made to formalize the role of structural trait diversity in forest ecology and evolution with the development of a ‘plant structural economic spectrum’ to complement the existing ‘wood economic spectrum’ and ‘leaf economic spectrum’ (Verbeeck *et al.*, 2019).

Structural diversity is generated through spatially varying factors that determine the availability of microhabitats within the area, setting preconditions for the existence of different species with varying niche requirements (Svenning, 1999; Dymytrova *et al.*, 2016). Spatially varying microhabitats arise from factors such as small-scale variation in soil properties (Matkala *et al.*, 2020), landscape topography (Zuleta *et al.*, 2020), hydrological features of the terrain (Francis *et al.*, 2020), and the inflows and outflows of biologically active material (including plants, animals, fungi, microbes, nutrients) to and from the forest of interest (Honkaniemi *et al.*, 2021).

Further structural diversity is generated (1) through dynamic 3-D growth and development of woody plants and other herbaceous vegetation within a forest stand and (2) through constantly changing environmental factors, such as weather, and a wide choice of unpredictable anthropogenic and natural disturbances (land use, global warming, wind, fire, snow, pest outbreaks, landslides etc.), as well as (3) through temporal changes in the inflow and outflow of biologically active material. The growth of woody plants alone causes temporal changes, because it alters individual plant architecture (3-D spatial arrangement of the plant structure), creating within-plant structural diversity through microenvironments within branches and foliage, i.e. due to different structural features (Escudero *et al.*, 2017; Asbeck *et al.*, 2019), self-shading (Ventre-Lespiaucq *et al.*, 2016) or weather (Nock *et al.*, 2016). Heterogeneous microenvironments inside a crown can thus alter growth habits (Koyama *et al.*, 2020) and the functioning of foliage (Ngao *et al.*, 2017; Curtis *et al.*, 2019). Growth of individual plants changes the spatial structure of the whole forest stand (e.g. canopy layers, canopy cover, gap dynamics, tree size variation), and, in combination with the flow of dead material from woody plants to the ground, generates further changes in the structural diversity and choice of microhabitats available (Kaufmann *et al.*, 2018). This results in the well-known natural succession of forest stands over decades (Boukili and Chazdon, 2017; Hilmers *et al.*, 2018). In yet longer temporal scales, this results in evolution over centuries to millennia as biological adaptations to changing and often unpredictable growth conditions appear (Areces-Berazain *et al.*, 2021).

Given all the constituents of structural diversity, it is clear that representative sampling of dynamically changing individual woody plant and forest characteristics is challenging. Even at the scale of individual woody plants, for instance in studies of the growth and architectural form of particular species, it can be challenging to explain the observations without information about the spatial structure of the growing site (Lintunen and Kaitaniemi, 2010; Kunz *et al.*, 2019; Hildebrand *et al.*, 2021), about the architectural structure and functioning of the individual itself (Posada *et al.*, 2009; Kennedy 2010), and without information about the temporal dynamics that are likely in the typical growth environment (Ishii *et al.*, 2018).

Traditional forest research can be labour-intensive and time-consuming. Detailed structural measurements of individual woody plants across a large stand have often been considered impossible and, instead, allometric or other structural relationships are used as proxies for multiple structural traits (Condés *et al.*, 2020). Destructive sampling or special climbing constructions have been essential to reach the top of large trees (Barker and Pinard, 2001). In addition, a long time-series beyond a typical researcher’s career would be required to capture the full dynamics of individual and stand development over time.

Now there is a potential game changer: a combination of terrestrial laser scanning (TLS) and highly detailed functional–structural plant models (FSPMs) that mimic actual plants (e.g. Louarn and Song, 2020) Together, they provide a pair of tools that fulfil many prerequisites for addressing challenges with research questions related to the structural dynamics of forests. Terrestrial laser scanning, optionally complemented with scanning from unmanned aerial vehicles, is a method that enables capturing the full 3-D structure of a forest stand with close to centimetre-level detail in a short amount of time (Liang *et al.*, 2016; Calders *et al.*, 2020), including the identification of individual trees and their species (Xi *et al.*, 2020), the ground vegetation (Muumbe *et al.*, 2021) and the topography of the terrain (Diamond *et al.*, 2020). Methods for collecting additional spectral information to estimate the state of various physiological processes within trees along with the structural scanning are also being developed (Junttila *et al.*, 2021).

**Fig. 1.**
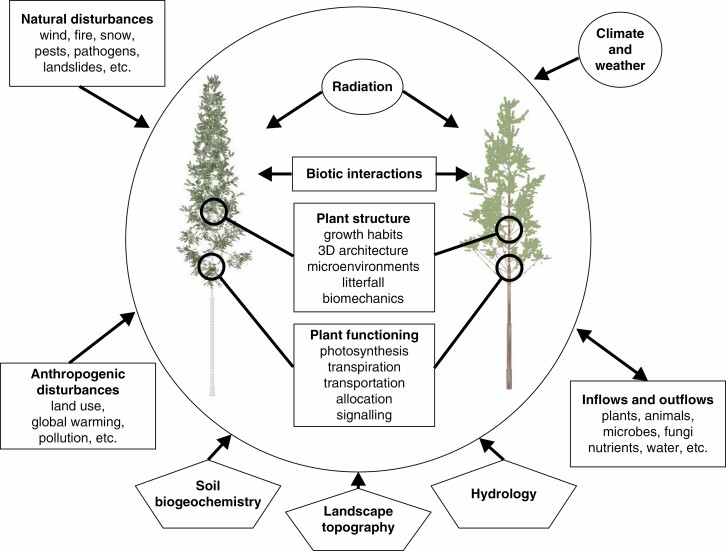
Structural heterogeneity of forest vegetation results from the interplay among multiple factors that influence structure and functioning of individual plants within forest stands over space and time. Current FSPMs largely focus on the encircled core of the system and consider the external factors with varying detail.

Functional–structural plant models, in turn, are models that aim to integrate the dynamic development of 3-D plant architecture, the associated physiological functions of the plant, and the simultaneous influence of external factors present in the growth environment (Fig. 1). As they operate with the 3-D architectural form of plants and plant stands, FSPMs are suitable for exploring the effects of different constituents of structural diversity on the dynamic behaviour of ecosystems. However, the applicability of FSPMs has so far been limited, much due to the amount of required data and high computational demands of the models; hence the existing models typically cover the spatial extent of just a few tree individuals (Sievänen *et al.*, 2008; Zhang and DeAngelis 2020). On the other hand, one of the former limitations in the development of FSPMs, the availability of structural data for the construction and validation of models, is now changing as FSPMs are suited to directly utilize data provided by TLS, and use it to develop models that explore long-term dynamic processes within trees and stands (Sievänen *et al.*, 2018).

In this review, we will focus on recent developments of TLS and FSPMs, evaluate their current capabilities in data collection and modelling capacity, and evaluate the current and anticipated future applicability of these techniques in solving ecological and evolutionary questions when used in combination. In the first of the following three sections we will address the impact of plant architecture on vegetation modelling from the individual plant level to the forest level and the impacts of forest evolution. In the second section we will explore FSPMs and their three different sub-models: (1) physiological, (2) environmental and (3) architectural. In the third section we will evaluate the realm of TLS and the benefits that this field has to scale up FSPMs from an individual-level modelling paradigm to a community-level modelling paradigm. In the third section we also propose a potential TLS to FSPM workflow, evaluating the advantages and disadvantages of data collection and software involved. Lastly, we discuss the current and future synergies between TLS and FSPM for modelling forest communities.

## MODELS OF INDIVIDUAL WOODY PLANT STRUCTURE AND FUNCTION

The form and function of woody plants, primarily trees, has captured the attention of biologists, mathematicians and artists alike for centuries. Since Da Vinci’s first mathematical formalization of tree structure, a large body of work has been dedicated towards understanding the mechanisms responsible for the array of tree shapes found across different habitats (Richter, 1970). Woody plants are dynamic organisms, composed of numerous self-similar and semi-autonomous parts, continually growing and developing from germination until death (White, 1979). In an effort to better understand the multitude of potential shapes, two botanists, Hallé and Oldeman, developed a qualitative system of describing different tree architectures found throughout tropical forests (Hallé *et al.*, 1978). The concept of tree architecture eventually gave rise to the discipline of ‘architectural analysis’, which aims to investigate how underlying genetics and environmental adaptation contribute to ultimate tree form and function (Barthélémy and Caraglio, 2007).

Tree form and function have been studied using various hypotheses on how the extension growth, space-filling by the crown and thickening of already existing woody axes and root growth and the assimilation yield are related (West *et al.*, 1999; Pałubicki *et al.*, 2019). For example, regular scaling between woody axes and growing shoots or leaves has been observed and explained by several theories/models (Shinozaki *et al.*, 1964; West *et al.*, 2009). Such detail is sufficient for accurate characterization of crown and tree development (Sterck and Schieving, 2011), but since the physiological mechanisms for the processes are only partially known, the approach needs to rely on empirically determined or theoretical principles. Further, the parameter values of the scaling relationships vary with growing conditions and with tree crowns (Bentley *et al.*, 2013).

Other approaches have also been used in the investigation of woody plant form and function: context-sensitive rules of development in crown dynamics can be applied that are related to physiology, including a carbon–nitrogen balance (Valentine and Mäkelä, 2012), photosynthesis (Peltoniemi *et al.*, 2012) or transport of carbohydrates within the crown (Nikinmaa *et al.*, 2013). Alternatively, rules can be defined by self-organization in branch growth (Pałubicki *et al.*, 2009). It has also been argued that the crown development actually follows resource optimization (Givnish, 1984; Mäkelä *et al.*, 2008; Sterck and Schieving, 2011; Franklin *et al.*, 2020). Understanding the mechanisms of individual resource partitioning in woody plants and how this influences the 3-D shapes we see in nature underpins the overall structure of forests. One important area of active research is the accurate estimation of above-ground biomass (AGB) in forests globally, especially in the context of rapidly changing environments (Brienen *et al.*, 2015). This inevitably relies on how well carbon allocation towards the different woody plant organs can be quantified. Formalized tree allometric models have been widely used to estimate carbon stocks and balance in forests (Chave *et al*., 2005, 2014). Whilst this is important for practical management and conservation, predicting future states of individual trees and their relative contributions to AGB requires further investigation of the driving processes of individual form and function in the context of their respective communities.

### Models of forest communities

Unravelling the mechanisms of woody plant form and function in forest communities, across different habitats, is crucial for furthering ecological theory, designing forest management plans and informing global land surface models (Franklin *et al.*, 2020; Ruiz-Benito *et al.*, 2020). However, the precise mechanisms that determine form and function of woody plants in forest environments have been largely overlooked in ecology until recently (Malhi *et al.*, 2018). In addition to the barriers in collecting data on forest communities (i.e. the impracticality of dealing with large, long-lived individuals and destructive harvesting), which make woody plants less than ideal subjects for experimental investigation, there remain a number of barriers in how organisms with a sessile but highly adaptive growth habit are sampled. Many of the methods typically used in ecology, such as counting individuals of varying age and size class, are insufficient to explain the broad intraspecific and interspecific variation found in forests (Harper, 1977, 1980). Furthermore, community ecology research had been dominated by a ‘mean field theory’ approach for years, which emphasizes differences between co-occurring species rather than differences between individuals of the same species (McGill *et al.*, 2006; Weiher *et al.*, 2011).

It is only in the last decade that efforts have been made to revisit the importance of intraspecific variation as a driver of community assemblage and integrate it into community ecology (Violle *et al.*, 2012; Hildebrand *et al.*, 2021). This is particularly pertinent for forests, where potentially high levels of intraspecific variation but low levels of interspecific variation can occur, as individuals converge on a common limiting resource (MacFarlane and Kane, 2017). Comparisons between mean field theory approaches and spatially explicit individual-based models (IBMs) have indicated that the spatial distribution of individuals in a forest can better explain ecosystem function and biomass production seen in nature, as opposed to a mean-field approach at the community level (Pacala and Deutschman, 1995). Woody plants interact on a local rather than a global scale, and therefore individual functional variability and growth strategies have clear implications for models at higher levels of ecological organization, such as dynamic global vegetation models (DGVMs) (Scheiter *et al.*, 2013). Spatially explicit IBMs have been used for decades in plant and forest ecology and forest science to better address heterogeneity amongst individuals in a community (Houston *et al.*, 1988; Grime, 2006). These models are able to capture many key elements of plant competition, such as individual variation and heterogeneous distribution of resources (Fisher *et al*., 2017). However, they traditionally lack a mechanistic explanation of these phenomena (Canham *et al.*, 2004). Moreover, these models do not interpret how individuals modify their local environment through 3-D adaptive behaviour (Grime, 2006; Brooker *et al.*, 2008).

Moving away from ‘mean’ approaches, whether that be in practical vegetation modelling or the development of paradigmatic theories, is paramount to fully exploring the drivers of forest structure and dynamics. In summary, a model with an applicability for diverse studies of evolutionary and ecological questions involving woody plant communities should possess the following properties in order to account for the role of vegetation structure and function in the processes investigated: it should account for the main processes of plant development (Sorrensen-Cothern *et al.*, 1993; DeAngelis 2018): (1) resource capture as a response to the immediate environment; (2) allocation of growth that results from resource capture to the development of the 3-D crown architecture; and consequently (3) modification of the immediate environment, described as a 3-D distribution of the resource flux. The model should also have modules that are sufficiently easily parameterized for (4) physiological processes (growth engine) and (5) morphological development (crown architecture) for different species. It would be beneficial if (6) different plant parts (organs) could be singled out (e.g. node, internode, leaf, bud) without simplified representations, such as spatial distributions. Further, (7) the model should be able to cover the whole life span of the woody plant, from seedling to maturity, including some measure of fecundity and mortality. (8) Spatially explicit individual-based simulations of a sufficiently large plant community are necessary. (9) An easy link with structural measurements would be useful, and finally (10) the model should be able to incorporate temporal variation.

Numerous plant community models are capable of fulfilling at least some of the above criteria. For example, process-based models can describe growth derived from physiological processes interacting with the environment (criteria 2 and 4 above) and have been used successfully for many years in agronomy (Bouman *et al.*, 1996; Marcelis *et al.*, 1998) and forest sciences (Mäkelä *et al.*, 2000). However, they lack the 3-D spatial and structural information of individuals which ultimately modifies fine-scale resource capture and growth in forest environments. Alternatively, gap models (Botkin *et al.*, 1972) include detailed life cycles and can differentiate between different species (criteria 1 and 5), which is beneficial for modelling plant community interactions. However, neither the specific 3-D form of individuals nor their spatial distribution in a simulation (criteria 2, 3 and 8) is explicit. There also exist ‘cohort-based models’, which act as an intermediary between IBMs and DGVMs, whereby plants are grouped by age, size or functional types (Fisher *et al*., 2017). These models are not as computationally heavy as IBMs but reduce the resolution of functional diversity (Fisher *et al.*, 2010). The placement and specific architecture of individuals within a community and their relative distances to each other are important factors for determining the strength of interactions between neighbours. Individual woody plant structure determines forest community structure, but this is itself determined by the structure of neighbouring woody plants. Models without 3-D structural variation will not capture within- and between-plant microenvironments, therefore losing information regarding fine-scale patterns of form and function in heterogeneous environments.

Indeed, many community models have been developed which account for forest structure and spatial heterogeneity including SORTIE (Pacala *et al.*, 2011), LPJ-GUESS (Smith *et al.*, 2014), ED/ED2 (Moorcroft *et al.*, 2001) and FORMIND (Köhler and Huth, 1998). These models have been able to answer a number of ecological and evolutionary questions with regard to forest structure and dynamics, including the effect of shading and crowding on neighbours (Canham *et al.*, 2004). Nevertheless, all these models have a simplistic representation of 3-D structure, omitting explicit branching and growth asymmetry, which can impact the presence of microenvironments. Indeed, high-level vegetation models including landscape forest models (Mladenoff, 2004) and DGVMs (Prentice *et al.*, 2007) rely on assumptions derived from plant community models, and thus it is important to investigate and evaluate the relative 3-D structural contributions of individuals in forest communities and how these ultimately impact vegetation assembly and dynamics (Zhang and DeAngelis, 2020).

### Evolutionary models

Evolutionary woody plant and community models typically operate with simplified or purely theoretical communities, and often focus on identifying different adaptive strategies and differences in niche utilization that are suggested to enable the development and coexistence of diverse woody plant species (Lamanna *et al.*, 2014). In recent years, these models have included the performance of individual plants that are growing in spatially explicit positions within a model system (May *et al.*, 2015; Coelho and Rangel, 2018; Wickman *et al.*, 2019). An accumulating line of evidence suggests that there can exist multiple alternative functional–structural tree designs that share an equal value of a fitness measure (Dias *et al.*, 2020), which is in line with the ideas of Pareto optimal plant design (Farnsworth and Niklas, 1995; Conn *et al.*, 2017) and the neutral theory of biodiversity (Hubbell, 2001). For example, Falster *et al.* (2017) demonstrated that the inclusion of few mechanistic details in a spatial forest model enabled diverse competitive coexistence in a manner that closely resembled the situation where different plant species are functionally identical. Xiao *et al.* (2007) concluded that there is no single evolutionarily stable height strategy for a plant population. Kennedy (2010) showed that alternative branch morphologies can compensate for water stress while simultaneously maximizing carbohydrate gain.

While increasing realism may sound beneficial, it can also easily lead to an added amount of model uncertainty due to practical challenges in specifying numerous parameter values with sufficient reliability. Equal performance of alternative designs also suggests that increasing the level of detail beyond a certain point may not produce further changes in model predictions, if the model simply depicts these alternative designs. However, detailed analyses of the roles of different functional and structural traits can be warranted in situations such as the need to select plants with favourable trait combinations for specific purposes (Picheny *et al.*, 2017). Hidden differences between alternative trait combinations may also become realized if the environment changes.

Franklin *et al.* (2020) suggested that the potential problems of model complexity in vegetation models could be circumvented by applying only three evolutionarily justified organizing principles to predict individual variation: natural selection, self-organization and entropy maximization. There are already examples where self-organizing principles alone can produce a high diversity of tree forms (Pałubicki *et al.*, 2009) and simulate long-term dynamic development of forest vegetation over a wider topographically variable area (Makowski *et al.*, 2019).

## GENERAL FEATURES OF FSPMS

**Fig. 2.**
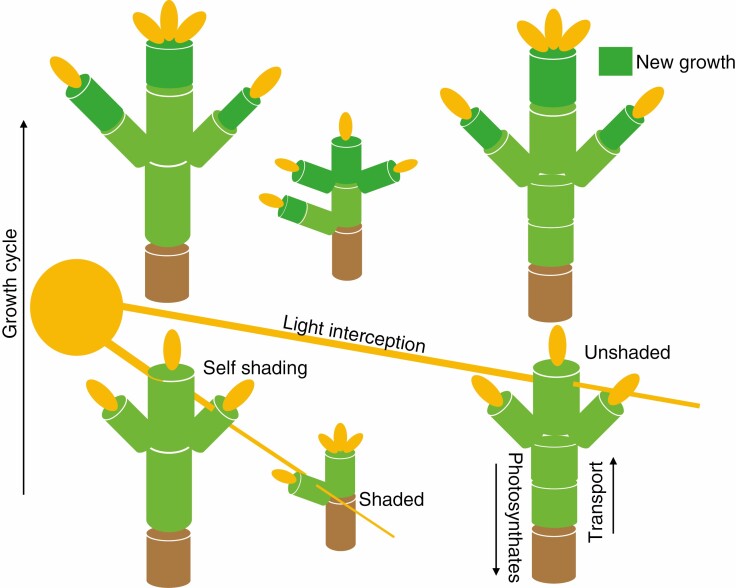
A schematic representation of how an FSPM interacts with the environment with multiple individuals simulated. Here, an environmental sub-model determines the amount of light available for photosynthesis. The overall structure of the plant determines how much light can reach different parts of the plant, which is then utilized by the physiological sub-model to calculate metabolites used for the next growth cycle. In multi-individual simulations, light is intercepted by neighbours, thus reducing resource capture.

**Fig. 3.**
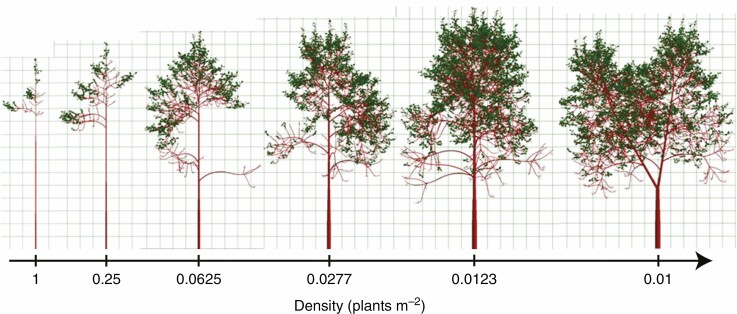
Example of FSPM function under changing environmental factors (Cournède *et al.*, 2008). In this case, the density of individuals decreases from left to right, highlighting the impact of growing conditions on tree structure.

Functional–structural plant models are complex 77models, but in light of increasing data availability, they now have the potential to be useful as tools for investigations on forest communities from the dynamic and evolutionary point of view and fulfil all the desired criteria outlined above (Louarn and Song, 2020). The FSPMs appeared in the mid-1990s with the aim of unifying classic process-based plant models with geometric models (Sievänen *et al.*, 2000; Godin and Sinoquet, 2005). FSPMs differ from earlier models of plant growth by considering an individual plant as a collection of semi-autonomous, interconnected elementary units. Metabolites allocated to those units are determined through availability derived from metabolic processes in the context of their environment (Fig. 2). The resulting model is structurally and functionally realistic and expresses highly responsive behaviour in relation to the prevailing growth conditions, which gives insight into the nuances of 3-D structure otherwise overlooked in other modelling techniques (Fig. 3). A good sample of previous research into FSPMs and related topics can be found in special issues of *New Phytologist***166** (3), 2005; *Functional Plant Biology* 35, 2008; *Annals of Botany***101** (8), 2008, **107** (5), 2011, **108** (6), 2011, **114** (4), 2014; **121** (5), 2018 and **126** (4), 2020; and *Ecological Modelling***290** (1–2), 2014.

Table 1 shows a sample of FSPMs applied for simulation of growth of woody plants. The most popular purpose of model construction is in the realms of horticulture and agronomy. This is understandable since the FSPMs are well suited for increasing understanding of resource flux to desirable products and simulation of management effects such as pruning. A clear driver in the development of these models is to improve our understanding of the interplay between resource acquisition and architectural development. Notably, these models are all based on resource capture of aerial parts only. Some models include roots but only as a passive part of the plant. There are numerous functional–structural models of root systems (Barczi *et al.*, 2018; Schnepf *et al.*, 2018) but none of them have been linked to the models listed here. Some of the models have been realized using a dedicated software for FSPMs. Notable examples of such software include AmapSim (Barczi *et al.*, 2018), GroIMP (Hemmerling *et al.*, 2008), Virtual Laboratory/L-studio, (http://www.algorithmicbotany.org/virtual_laboratory/; Prusinkiewicz, 2000) and OpenAlea/OpenAleaLab (https://team.inria.fr/virtualplants/software/; Pradal *et al.*, 2008). Amongst the many FSPMs that have been developed, significant variation can be found in their implementation. Nevertheless, FSPMs always feature three main components or sub-models: a physiological model, an environmental model and a structural model.

**Table 1.** An overview of FSPMs that simulate dynamic growth of woody plant species. In addition to the publications, information about the models on the site www.quantitative-plant.org has been utilized. N.i., no information available; UML, Unified modelling language

Model	Species	Time step/time range	Parameter input	Multi-plant simulation?	Programming language/software	Open-source	Purpose/comment	Citation
ECOPHYS	Poplar	Hour/several years	Biologically derived	Yes	C++	N.i.	Disentangling growth factors	(Host *et al*., 1996, 2008)
GreenLab	Generic/beech, Mongolian pine	Can choose/decades	Biologically derived/Stat. fitting	Yes	Matlab, Java, Cpp, Scilab	Upon request, freeware	Resource capture, plant architecture	(Cournède *et al.*, 2008; Letort *et al.*, 2008; Wang *et al.*, 2012)
GroIMP (Beech)	Generic/beech	Year/decades	Biologically derived	Yes	GroIMP, XL (Kniemeyer and Kurth, 2008)	Yes	Demonstration of software	(Hemmerling *et al.*, 2008)
IMapple	Apple tree	Hour/decades	Biologically derived	Yes	C#	N.i.	Horticulture	(Kang *et al.*, 2016)
LIGNUM	Pine, maple, poplar/generic	Year or growth cycle/decades	Biologically derived	Yes	C++, L+C^c^ (Vos *et al.*, 2007)	Upon request, GitHub	Study of crown development	(Perttunen 2001; Sievänen *et al.*, 2008; Lu *et al.*, 2011)
L-Peach	Peach tree	~1 d/several years	Biologically derived	Focus is one tree	L-studio (Prusinkiewicz, 2000)	Upon request	Horticulture	(Allen *et al.*, 2005; Lopez *et al.*, 2008; Da Silva *et al.*, 2014)
Macadamia model	Macadamia tree	Day/growing season	Biologically derived	No	L-studio (Prusinkiewicz, 2000), L + C (Vos *et al.*, 2007)	N.i.	Horticulture, proof of concept	(Auzmendi and Hanan 2020)
MAppleT	Apple tree	~1 d/several years	Biologically derived	Focus is one tree	L-Py (Boudon *et al.*, 2012), OpenAlea (Pradal *et al.*, 2008)	Yes	Horticulture	(Costes *et al.*, 2008)
QualiTree	Peach (fruit trees)	Day	Biologically derived	Yes (canopies as ellipsoids)	UML (object technologies)	N.i.	Horticulture, fruit development	(Lescourret *et al.*, 2011; Mirás-Avalos *et al.*, 2011)
Radiata pine	*Pinus radiata*	Month/several years	Biologically derived	No	Basic	N.i.	Silvicultural decisions	(Fernández *et al.*, 2011)
Sterck model	Generic deciduous	Day/decades	Biologically derived	No	N.i.	N.i.	Ecological–evolutionary problems	(Sterck *et al.*, 2005; Sterck and Schieving 2007)
V-Mango	Mango tree	Day/several years	Stat. fitting/Biologically derived	No	L-Py (Boudon *et al.*, 2012), OpenAlea (Pradal *et al.*, 2008)	N.i.	Horticulture	(Boudon *et al.*, 2020)

The main focus of the FSPM studies has been in the responses of single plants, either isolated or as a part of a plant community. If they have been part of a plant community, the effect of other plants has been modelled assuming some sort of homogeneity of the surrounding canopy. For example, Sterck *et al.* (2005) assume the surrounding canopy be characterized by its height and a homogeneous leaf distribution. Nevertheless, in spite of the predominant focus on individuals of FSPM tree applications, five of the 12 models listed in Table 1 are capable of simulating interacting tree individuals, i.e. scaling up from elementary units to tree community. Host *et al.* (2008) used the ECOPHYS model (Table 1) to simulate the growth over 8 years of individual aspen and *Populus* trees on a patch of 64 trees with an hourly time step. They observed how the growth differed between various clones for which the parameters of e.g. leaf-level light interception, carbon allocation, canopy architecture and phenological events had been determined. Cournède *et al.* (2008) simulated the effect of interplant competition for light and density on interacting individual trees in heterogeneous conditions using the GreenLab model (Table 1). Hemmerling *et al.* (2008) showed a simulation using the model GroImp (Table 1) of a landscape consisting of 700 individual beech and spruce trees for 11 years with an annual time step when the trees were interacting through radiation interception.

Computational requirements are no longer a major obstacle in the development of FSPMs at the community level. Taking the LIGNUM model (C++ implementation, Sievänen *et al.*, 2008) as an example of computational requirement of multitree simulation, a rough estimate is as follows. The elementary units tree segment and bud can take 300 and 200 bytes, respectively. A tree with 50 000 units will consume 25 megabytes of memory. Extending this to 3000 trees (per ha) the memory requirement would be ~75 gigabytes. The second memory requirement comes from environmental modelling. One way to represent the growth space of plants is to discretize it to cubic volume elements using voxel space. For example, in the LIGNUM model, the individual elementary cubic element (a voxel), requires ~300 bytes. Assuming a voxel with 20 cm side length (roughly the size of an elementary unit tree segment) and the requirement for a 1-ha forest plot 40 m high, the number of voxels would be 50 million. This amounts to a roughly 15 gigabyte memory requirement for the voxel space.

A modern high-performance computing system can have hundreds of gigabytes or even terabytes of available memory (see examples at docs.csc.fi computing environment). Therefore, the large number of elementary units does not create an insurmountable computational obstacle as long as physiological processes can be computed in linear time, *O*(*n*), in terms of elementary units, as is the case in most of the models in Table 1, including the calculation of light transmission in canopy or tree crowns, for example, with the aid of voxel space (Sievänen *et al.*, 2008). These examples indicate that the FSPMs are capable of simulating heterogeneous natural forests as interacting individual trees.

Major strides have been made in the physiological models, the environmental model and the structural model, particularly for non-woody plants, namely, the development of state-of-the-art light models (Dieleman *et al.*, 2019), below-ground extensions of 3-D architecture (Barczi *et al.*, 2018; Schnepf *et al.*, 2018) and numerous physiological advances (Louarn and Song, 2020). So far, FSPM development has been largely dedicated towards modelling crop products: for example, grapes (J. Zhu *et al.*, 2018; Schmidt *et al.*, 2019; Prieto *et al.*, 2020), tomatoes (Sarlikioti *et al.*, 2011; Dieleman *et al.*, 2019; Vermeiren *et al.*, 2020), soybeans (Coussement *et al.*, 2020; Song *et al.*, 2020). The development of FSPM components in agronomy has been pivotal to the extension and improvement of FSPMs in general (Vos *et al.*, 2010; de Reffye *et al.*, 2021), but careful consideration must be taken when applying these models to ecological and evolutionary questions. For example, many agronomy-based FSPMs target fruit production (Table 1) and thus include detailed source–sink components that may be unnecessary for modelling natural forest stands (Allen *et al.*, 2005). It is also worth noting that agronomy FSPMs often simulate growth and development in optimum environments, without any nutrient, water or light shortages (Auzmendi and Hanan, 2020), which is far removed from natural conditions. In short, each sub-model (environmental, structural and physiological) must be evaluated in turn to ascertain trade-offs between reality, generality and simplicity at larger spatial and temporal scales.

### Physiological sub-models

The modular nature of FSPMs means that almost any physiological models (empirical or mechanistic) can be included and exchanged depending on the research question. Whilst this has benefits in testing and exploring a range of theories, it can quickly become overwhelming for non-experts. The growth and development of woody plants can be split into two categories of physiological processes: the ‘fast processes’ of photosynthesis and respiration and the ‘slow process’ of carbon allocation. Carbon is assimilated through photosynthesis and allocated either towards new growth or maintenance, whilst carbon losses occur through respiration and senescence. Photosynthesis has been represented in FSPMs in a number of ways, including implicit models (Mäkelä and Hari, 1986), empirical models (Le Roux *et al.*, 2001) and biochemical models, notably the Farquhar–von Caemmerer–Berry (FvCB) model for C3 photosynthesis (Farquhar *et al.*, 1980; von Caemmerer and Farquhar, 1981), which has been scaled up for whole canopies in Medlyn *et al.* (2003). Biochemical models have the added benefit of representing instantaneous responses at small time steps of minutes and hours, which may be beneficial for particular studies, for example in modelling fast-growing species (Lu *et al.*, 2011). However, these short physiological time steps may not be necessary, or appropriate, for modelling forest structure and dynamics at larger spatial and temporal scales.

Notably, most of the FSPMs listed in Table 1 either ignore acclimation or prescribe fixed parameter values for each specific species. However, vascular plants are known to adapt their ‘fast’ physiological processes depending on their environmental conditions (Walters, 2005). For photosynthesis, a mechanistic approach, such as that employed by the P-model (Stocker *et al.*, 2019) may be more appropriate for simulating the acclimation of photosynthesis over time. Here, an optimality-based theory is used to predict the acclimation of leaf-level photosynthesis to its environment. Moreover, a recent global analysis of forest production efficiency by (Collalti *et al.*, 2020) observed that respiration rates varied with temperature far less steeply than observed in short-term responses, indicating acclimation over time. ‘Fast’ physiological processes in FSPMs are usually based on these instantaneous responses and it will be important in future development to consider the impact of this aspect on simulations at the scale of weeks, months and years. In the FSPM modelling paradigm, which is based on the concept of a plant’s adaptability to its local environment, it is imperative not to underestimate the importance of acclimation in basic physiological responses. Some areas to consider are CO_2_ acclimation as implemented in mCanopy-soybean (Song *et al*., 2020) or the light acclimation simulations of sugar maple trees in LIGNUM (Posada *et al.*, 2012).

The ‘slow’ process of carbon allocation towards new growth and maintenance of existing organs is particularly important in FSPMs, but also one of the least well understood. Carbon allocation methods are difficult to compare between FSPMs because different models employ different groupings of parts (Zhang and DeAngelis, 2020). Most FSPMs include a carbon allocation model based on the pipe model theory (PMT) proposed by Shinozaki *et al.* (1964) over half a century ago (Godin, 2000; Le Roux *et al.*, 2001; Lehnebach *et al.*, 2018). Whilst this model provides a useful framework for investigating functional–structural relationships, many of its assumptions are outdated. Notably, in their comprehensive review of the PMT, Lehnebach *et al.* (2018) find that the property of sapwood area preservation is almost never valid, and that the ratio of leaf mass to a cross-sectional area of sapwood can vary greatly depending on internal and external properties. As an alternative, the authors suggest an additional model step not included in the PMT, which robustly quantifies foliage and sapwood partitioning regardless of species, age or stand condition. A broad uptake of such methods could be useful for comparing between FSPM models, particularly with regard to how carbon allocation is represented in models of herbaceous and woody plants. Furthermore, it permits explaining potential physiological adaptations found between individuals in a given forest community. It is worth noting that physiological sub-models of FSPMs are one of the greatest contributors of model complexity and still one of the most difficult to parameterize effectively, so a move towards simpler physiological sub-models would be greatly beneficial to FSPMs, particularly when applied to natural, multitree scenarios.

### Environmental sub-models

Simulations of realistic local environmental conditions are a key determinant of how woody plants adjust to external processes. In FSPMs there are many ways to represent environmental conditions and these methods come with varying levels of reality and detail. A select few FSPMs have been able to capture detailed below-ground processes in herbaceous plants, notably C-Root Box (Schnepf *et al.*, 2018), but the majority of FSPMs focus on above-ground processes. Thus, in terms of resource capture, light conditions are fundamental to understanding how individuals optimize form and function within their respective communities. Recent FSPM studies have indicated that light competition reduces individual plant defence against herbivory (de Vries *et al*., 2018, 2019). In addition, Douma *et al.* (2019), have shown that it is not only the quantity of light that contributes to plant defence, but also the quality of available light.

Thus, modellers must evaluate the potential options for representing an accurate light environment when developing FSPMs and scaling up from organ level to a forest patch by simulating the growth of individual trees. As the development of tree individuals takes place in terms of a collection of elementary units without simplifying assumptions of crown shape (if it was, it would be contrary to the idea of FSPMs), it is not easy to make use of stand-level simplifications in the calculation of radiation conditions. Rather, the analysis of radiation conditions needs to be made on the basis of actual positions of all individual shading units (foliage) and it is a computationally complex problem (Host *et al.*, 2008). Although computers have advanced rapidly in recent years, the choice of light model can add a significant computational burden to the overall FSPM so trade-offs are inevitable.

Most methods of light simulation involve computing how much photosynthetically active radiation (PAR) is intercepted or absorbed by plant structural elements. To speed up radiation calculations, spatial discretization, such as the method of voxel space (Li *et al.*, 2018), or some other means, like techniques based on Monte Carlo sampling in path tracing (Cieslak *et al.*, 2008), can be applied. Alternatively, shadow propagation (Pałubicki *et al.*, 2009) has been used, which is a fast approach to compute a coarse estimate of the exposure of each bud to light. In this approach the space is divided into a grid of voxels each with associated shadow value. Each plant segment occupying a particular voxel creates a pyramidal penumbra at the voxels underneath. All segments are sequentially processed to produce a 3-D grid of accumulated shadow values.

More recently, with improved parallel processing and the use of graphics processing units (GPUs), it has become possible to model detailed absorption, transmission and reflectance for the full light spectrum using ray-tracing models (Henke and Buck-Sorlin, 2017). A normal forward ray-tracing is based on the point of view of the radiation source (sky). This means that the rays from the source are traced to the elements of interest (i.e. leaves, buds, branches). However, this approach becomes problematic when the number of elements becomes large, because to sample them sufficiently and accurately requires a very large number of rays that sample the incoming radiation. Even then, there can be large errors in individual elements. The idea of reverse ray-tracing is to change the point of view from the radiation source to the entities themselves. Thus, the rays from every element are traced to the source, which ensures that each element is sampled properly and the errors are bounded. This approach has been used in a number of static FSPMs, including Helios (Bailey, 2018, 2019), and the FSPM generation platform GroIMP (Kniemeyer and Kurth, 2008; Hemmerling *et al.*, 2008).

### Architectural sub-models

One of the most widely known and commonly used methods of representing the 3-D structure of woody plants is through Lindenmayer systems, otherwise known as L-systems (Prusinkiewicz, 2000). L-systems are parallel rewriting strings that act on sequences of symbols. Woody plants can be represented by an L-system with an alphabet of symbols and a set of rewriting rules, known as productions. Each symbol in the alphabet represents a distinct botanical unit, such as buds, leaves or flowers. This system of rewriting allows for dynamic growth rather than just a static configuration of plant architecture in space. Numerous FSPMs use L-systems directly or software based on them, such as L-studio, L + C and L-Py (Table 1) to define plant structure. Creating successful L-systems that reflect realistic plant forms is an art in itself (Prusinkiewicz, 2004). It can quickly become laborious when multiple species with different growth habits, or individuals of different ages, need to be defined. Parameterizing L-systems in an informative way requires many structural parameters, which were difficult, if not impossible, to attain until the recent application of TLS technology to forest communities.

## THE REALM OF TLS

In recent years, TLS has emerged as a revolutionary tool for measuring above-ground 3-D tree architecture and forest structure (Malhi *et al.*, 2018; Disney, 2019; Calders *et al.*, 2020). TLS is a ground-based remote sensing method of determining distance and is one of a wider choice of laser scanning techniques that provide often complementary information for different measurement scales and research purposes (Beland *et al.*, 2019). Here, we consider TLS to also cover scanning from unmanned aerial vehicles capable of accessing the upper parts of the canopy (Slavík *et al.*, 2020). There are numerous TLS instruments available commercially, and whilst they vary greatly in their specifications, they all function in the same way, by emitting a laser light onto a 3-D object and measuring the distance to the object. Three-dimensional representations, called point clouds, can then be generated based on the distances and directions of the laser beams. The potential benefits of this technology for elucidating forest structure and dynamics can hardly be overstated. TLS is a non-destructive technique that can produce measurements to within even millimetre accuracy, depending on the equipment used, without the need to fell trees. This significantly reduces the time required for taking detailed measurements of individuals and leaves the habitat intact for potential resampling at a later date. As TLS technology matures, so does the ability to scan larger areas with excellent resolution. The large quantities of high-accuracy structural data provided by TLS are invaluable for elucidating the underlying mechanisms of individual tree form and thus the whole-forest organization. Despite the promises and potential, the application of TLS to questions in forest ecology and evolution is still in its early days. In a recent review, Malhi *et al.* (2018) outline a number of areas suitable for testing and extending ecological theory on tree form and function in the context of TLS, including seed dispersal, structural mechanics and resource distribution. The opportunities are indeed exciting; however, tackling such theoretical questions with TLS requires careful coordination of elements spanning many disciplines.

In practice, TLS is time-efficient in relation to the gained precision and is easy to use; however, there are a number of nuances that must be taken into consideration to ensure overlap of practice between researchers. Some challenges will be present regardless of the equipment used or environmental conditions, which include, but are not limited to, occlusion, leaf and wood separation, irregularity of form and validation. Many of the commercially available TLS instruments are able to reduce common difficulties associated with scanning individuals, stands and large areas of mixed-species forests. The potential to test and further expand ecological theory stems largely from the ability to gain an accurate representation of how organs of a tree are arranged in 3-D space and how multiple trees in this space are arranged relative to each other. Similar to choosing the correct environmental and physiological elements to include when creating FSPMs, correctly planning both the data collection and data processing prior to beginning a project is key to maximizing the benefits of TLS. If all the trees in a forest plot are targeted, Wilkes *et al.* (2017) suggest a sampling grid of 10 m × 10 m in dense areas and 20 m × 20 m in open areas to produce a good-quality point cloud that effectively resolves higher-order branches down to a few centimetres in diameter in up to 30 m canopies. If individual woody plants are targeted, the sampling grid can be designed on a case-by-case basis to maximize the visibility of the individual.

**Fig. 4.**
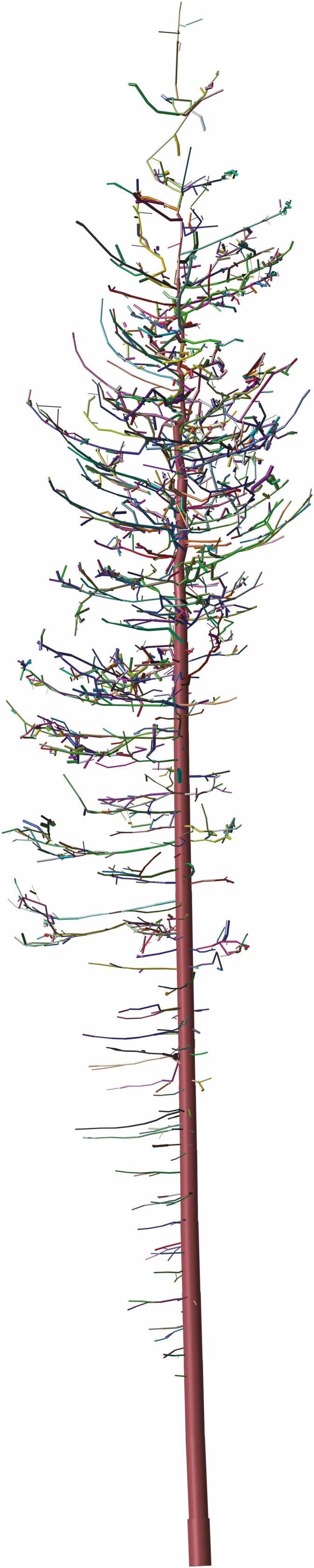
Example of a quantitative structural model (QSM). QSMs are vital for extracting detailed structural measurements of woody plants useful in downstream analysis such as FSPM input or validation and calibration. Cylinders are used to reconstruct 3-D structure from point cloud data. Here, the different colours represent the different branches of the tree.

A highly important use of TLS has been in quantifying AGB. Estimating AGB is crucial to our understanding of carbon stocks and fluxes but has been plagued by systematic error due to the difficulty in obtaining manual measurements, bias towards temperate regions and an over-reliance on allometric scaling equations. New TLS-derived measurements have deconstructed previous assumptions about AGB estimates and challenged the validity of many of the widely used allometric scaling equations (Disney *et al.*, 2018). Notably, TLS estimates of AGB are free from the constraints of allometric models and can thus provide well-justified levels of uncertainty. Estimates of AGB derived from TLS tend to perform well in a number of scenarios, including tropical and urban environments; however, it should be noted that it is not a mature enough tool to be used as a standalone methodology (Wilkes *et al.*, 2017; Lau *et al.*, 2019). Currently, measurements from destructive harvests are frequently required to validate AGB estimates from TLS. It is also noteworthy that allometric models can be refined towards more flexible functional models using the type of data that is available from TLS (Kaitaniemi *et al.*, 2020; Kaitaniemi and Lintunen, 2021). Alternatively, quantitative structural models (QSMs), which are a hierarchical collection of cylinders (Fig. 4), can be used to compute an array of detailed structural tree metrics, including AGB. There are published and freely available methods to reconstruct QSMs from TLS data; these include *TreeQSM* (Raumonen *et al.*, 2013; Calders *et al.*, 2015*a*; Raumonen, 2020), *SimpleTree* (Hackenberg *et al.*, 2015), *AdTree* (Du *et al.*, 2019) and *CompuTree* (Piboule *et al.*, 2013). *SimpleTree* is currently known as *SimpleForest* and it is a plugin for *CompuTree*. The accuracy and level of details available in the QSMs is heavily dependent on the quality of the input point clouds. For example, if a tree is sampled poorly such that a lot of branches are completely missing in the data, then these methods cannot reconstruct the missing branches.

**Fig. 5.**
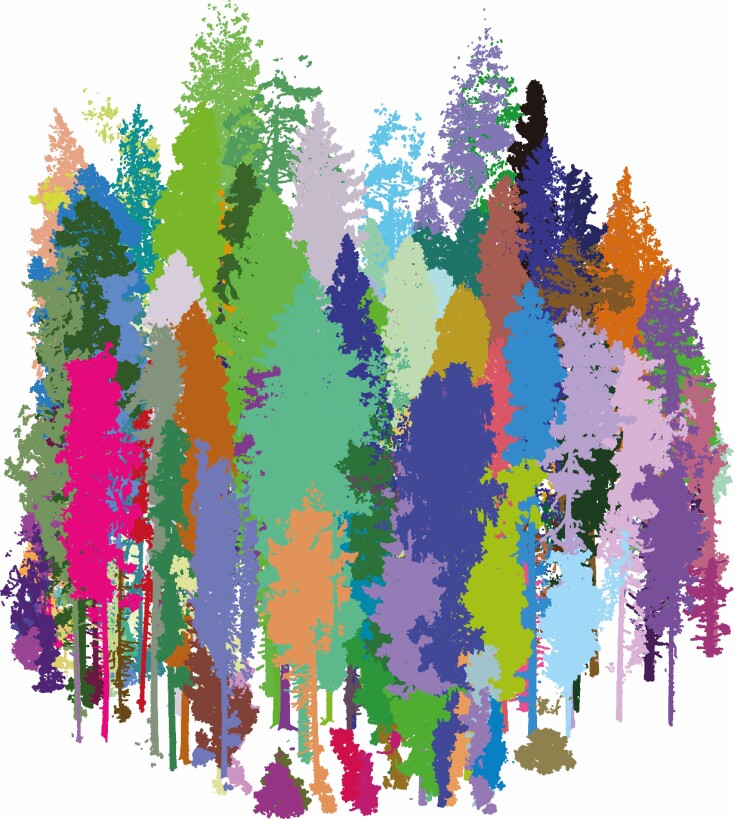
Segmentation of individual trees from a whole-forest scan. Although manual segmentation is possible, automation or semi-automation of this process can greatly speed up the processing of TLS data. The different colours delineate between individual trees in the forest community.

Differentiating between individual trees remains a key challenge when working with scans containing multiple trees. Whilst this is simple enough to do manually using open-source software such as CloudCompare, it becomes increasingly time-consuming relative to the size of the area covered as well as the density and structural detail of trees. Automated or semi-automated methods can help to streamline this area of TLS data processing (Fig. 5). However, error propagation arising from TLS automated workflows is a known issue and human assistance should be used where appropriate (Martin-Ducup *et al.*, 2021). There are at least two open-source software options available that tackle this issue in different ways. Firstly there is *treeseg* (Burt *et al.*, 2018) and *3dforest* (Trochta *et al.*, 2017), both developed with C++, which use a number of standard point cloud processing techniques; and secondly there is *LidR* (Roussel *et al.*, 2020), written in the R environment, intended for use with airborne LiDAR, but easily applied to TLS. These software options are invaluable for reducing the amount of time for point cloud processing in forest environments. However, due to the popularity of the R language in biological sciences compared with C++, *LidR* has the added benefit of accessibility.

Extensive point clouds of multiple trees become even more difficult to handle when they include different materials (i.e. leaf and wood) (Côté *et al.*, 2012). It is often recommended to complete scanning campaigns in leaf-off conditions but there are many scenarios where that may not be possible, or even desired, such as when calculating leaf area index or leaf area distribution. Therefore, a method of separating leaf and wood from point clouds is essential for working with TLS data in an ecological context. Indeed, calculations of leaf area index can be overestimated if woody material in the canopy is unaccounted for (Woodgate *et al.*, 2015). Similarly, woody AGB estimates can be overestimated if leaf points are unaccounted for. Methods for separating leaf and wood fall largely into two groups: geometric-based separation and intensity-based separation. Intensity-based methods use the measured intensity of the laser beam as a marker of the material, using a simple threshold value for the classification. However, this kind of simple approach does not perform well in general. The intensities may still be useful for classification as one of the information sources (X. Zhu *et al.*, 2018). Geometry-based methods compute multiple geometrical features characterizing each point’s neighbourhood and then train a leaf–wood classifier based on those features. With the variety of approaches available it can be daunting to select an appropriate method for a particular study. More recently, a method has been described with a high level of automation as well as options for flexibility. *TLSeparation* is an open-source Python library offering custom workflows and automated scripts (Vicari et al., 2019). This software performs with a 90 % accuracy for separating leaf and wood in field TLS data, which is a beneficial step towards efficient TLS data processing of large forest scans. There are other geometric-based published methods with similar accuracies (Wang *et al*., 2018, 2020; Moorthy *et al.*, 2020).

### Species identification

An area of TLS research with exceptional benefits to ecological modelling is the development of automatic species identification algorithms. A handful of studies have investigated potential methodologies in different scenarios. Othmani *et al.* (2013) used bark texture to identify five species from a sample size of 75 individuals using a random forest classification with an accuracy of 85 %. While this method can be useful for identifying species that are mostly different only in their bark, the required accuracy and resolution levels require close-range scanning. Barmpoutis *et al.* (2018) classified four species of Caatinga trees using the fast-marching method for tree skeletonization, subsequently classifying skeletons with descriptors that account for a combination of dynamic, appearance and noise parameters. Whilst this method outperformed alternative approaches based on geometric characteristics, the sample size (15 individual trees) is too low to determine whether or not it would be feasible to apply this methodology to large forest scans with lots of individuals.

Some success has been found with large sample sizes in studies using QSMs to obtain structural features and machine learning for classification. Åkerblom *et al*. (2017) obtained an average classification accuracy of >93 % in a single-species forest plot, with lower accuracy found in mixed-species plots. A more recent study by Terryn *et al.* (2020) expanded on this work using a mixed species plot of 760 trees across a 1.4-ha study area to compare classification. They were able to achieve a classification success rate of 80 %, but with lower accuracies for three out of the five target species. High intraspecific variation alongside low inter-specific variation led to classification complications, which are difficult to overcome in mixed-species plots. The authors find that the greatest factor contributing to classification success is canopy class. Whilst these results are not entirely surprising, they do exemplify the frustrating difficulty in applying quantitative techniques to organisms with high levels of local adaptation. Moreover, Martin-Ducup *et al.* (2020) found a convergence of tree architecture with increasingly dominant crown canopy positions in tropical trees. They interpreted this convergence as resulting from a liberation effect of canopy trees from side-shading constraints. These results also point to the challenges of using structural metrics for species identification. Further research is required in this area as FSPMs require species-specific information to function reliably, and any ability to automatically identify species across large areas would greatly improve the potential to apply FSPMs to larger ecological questions.

### TLS in studies of vegetation dynamics

TLS data, often with derived QSMs, have been used to gain new understanding of forest dynamics and structure. For example, Seidel *et al.* (2011) showed how crown plasticity in terms of crown asymmetry is used by trees to avoid competition. They concluded that TLS offers an opportunity to achieve a better understanding of the dynamics of canopy space exploration, which can produce valuable advice for the silvicultural management of mixed stands. Kunz *et al.* (2019) studied the crown plasticity more thoroughly by scanning thousands of trees in large-scale forest experiment plots with variable species mixtures with TLS. They found that crown complementarity and crown plasticity increased with species richness. Furthermore, trees growing in more species-rich neighbourhoods showed enhanced above-ground wood volume in trunks and branches, and over time the diversity induced shifts in wood volume allocation in favour of branches. Martin-Ducup *et al.* (2018), in turn, found that the foliage distribution of sugar maple shifted towards the crown base in mixed stands when compared with pure stands. Recently, van der Zee *et al.* (2021) introduced a novel method of quantifying the phenomenon of ‘crown-shyness’ by applying a 3-D surface complementarity metric to TLS data. With this method they were able to exemplify local adaptive responses in neighbouring trees and the relationship between crown complementarity and individual ‘slenderness’ mirroring previous results. Applying TLS in a functional context, Hildebrand *et al.* (2021) found no support that functional dissimilarity promoted crown complementarity in a mixed-species forest stand. Rather, they find that the relative efficiency of different species in occupying canopy space is better explained by phenotypic changes related to crown morphology and branch plasticity. This has interesting implications for how we view woody plant interactions, highlighting the need for a thorough 3-D approach to forest community ecology.

The phyllotactic patterns of many herbaceous plants are generated by a constant divergence angle between successive buds that first appears at the shoot. Beyer *et al.* (2021) investigated whether the branches along tree trunks exhibit a similar constant divergence angle. From branch skeleton data derived from TLS data they empirically estimated the distributions of the divergence angles between successive branches along the trunks. They found that, rather than having species-specific branch divergence angles, mature European beech, Norway spruce and Scots pine trees feature statistical properties characteristic of a uniform distribution. They hypothesized this to be the result of the stochasticity in bud development and branch shedding, and showed that the distribution of branch divergence angles will approximate a uniform distribution if bud mortality and branch shedding rates are high.

Calders *et al.* (2015b) monitored the timing of recurring seasonal dynamics through the plant area index (PAI). From TLS data they estimated vertical plant profiles, which describe the plant area per unit volume as a function of height. They generated a time series from 48 measurement days in a deciduous forest and the start of season was observed to depend on the species composition. They concluded that phenological differences will be more pronounced in multi-layered forests, and TLS was shown to have the potential to study seasonal dynamics not only as a function of time, but also as a function of canopy height. An exciting advancement in the area of TLS time series for monitoring forest dynamics is described by Campos *et al.* (2021), where they outline the creation of an automated and permanent TLS measurement station. This new monitoring station is the first to collect long-term TLS data at 1-h intervals. In preliminary results they were able to identify a number of short-term and long-term changes in a boreal forest, notably the short-term circadian rhythms in silver birch trees over 30 h, and longer-term phenological chances of spring leaf-sprout and stem diameter growth.

A number of studies have used TLS to investigate relationships between environmental factors and woody plant structure. TLS data and the derived QSMs of trees have been used for mechanical modelling of the trees under critical wind speeds (Jackson *et al.*, 2019). Mechanical stability is a vital component of woody plant form and function and here it was found that the difference in critical wind speed is driven by tree size and architecture, rather than material properties. In this study a trade-off between critical wind speeds and growth rate was also observed. Van der Zande *et al.* (2010) combined TLS measurements with a voxel-based light interception model to examine the relationship between variable light conditions and the distribution of leaves in 3-D space; their results exemplify the impact of neighbourhood crown size on the available light in the canopy.

### Using TLS to parameterize FSPMs

**Fig. 6.**
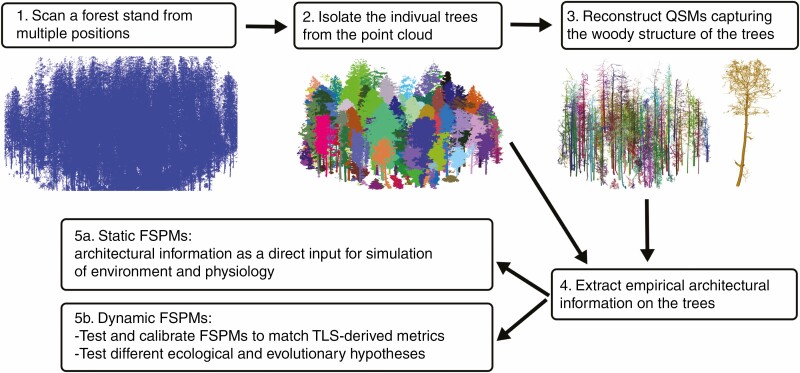
A potential TLS to FSPM workflow. 1. To minimise occlusion and gain an even point coverage, individuals should be scanned from multiple positions at regular intervals (see Wilkes *et al.* 2017). 2. Trees can be segmented from the large scan using segmentation software, LidR (Roussel *et al.* 2020) treeseg (Burt *et al.* 2018) or 3DForest (Trochta *et al.*, 2017). 3. Generate QSMs for each individual in the scan (Raumonen *et al.* 2013) 4. Extract the appropriate structural information for use in FSPMs. 5a. Structural information can be used directly to simulate function with static woody structure and changing environmental conditions. 5B. Alternatively, structural information can be used to initialise FSPMs, aid validation and calibrate dynamic models with changes in woody structure.

Once the individual trees are isolated from the TLS point cloud, and the points from leaves are removed if necessary, it is possible to calculate many useful structural metrics. Straightforward calculations are available for macroscopic metrics, such as height, crown diameter and convex bounding volumes. But more specific metrics, such as diameter at breast height, stem volume, crown ratio and branch lengths and volumes, require more processing, often some kind of segmentation of the point cloud into suitable tree parts. An effective way of reconstructing the whole woody structure is through the implementation of QSMs. The use of QSMs generates possibilities to use TLS data for FSPMs (Fig. 6). Firstly, simulating physiology and environment in a static context can directly use empirical tree models derived from TLS data. Secondly, these empirical structure models are useful for providing data needed to validate and test the 3-D structure of FSPMs against empirical data in order to estimate their accuracy and usefulness more generally. For example, measuring detailed and complete structural information, such as branching topology and diameters, of large trees with manual measurements is very laborious or even practically impossible. With TLS it is possible to quickly collect such data from a large number of individuals that form a representative sample. This often requires that the TLS data are first transformed into QSMs, from which the structural tree data can be computed and inferred. However, much of the required structural information can be estimated from TLS data without full QSMs, namely overall measures such as crown dimensions and woody plant height. Moreover, useful information about the leaves, such as total leaf area (Béland *et al.*, 2011) and leaf orientation (Zheng and Moskal, 2012) together with their spatial distributions, can be estimated from TLS data. Thirdly, the non-destructiveness of TLS allows for repeated measurements over a span of time, for example over a growth season or many years. This way it is possible to produce an empirical time series of 3-D architecture and sample growth dynamics. These time series can similarly be used to validate and test the accuracy of the dynamic modelling of woody plant growth in a given FSPM. Lastly, time series data are useful for initialization, calibration or optimization of the FSPM parameters to make them correspond better to the observed form and function of woody plants.

A concrete example of using TLS data for parameterization and validation of FSPMs, at a proof-of-concept level, was given by Sievänen *et al.* (2018). In this study, pine trees of different ages but in the same location were scanned with TLS. This created a pseudo-time series of growth of a single tree. From the TLS data, empirical cylindrical QSMs were reconstructed to model the tree architecture at each age interval of the tree. To complete the models with needle information, the location of needles was also estimated from the TLS data and together with needle allometry their area was estimated. The empirical tree models were thus similar in form as in LIGNUM. Then within the LIGNUM model many different crown development mechanisms were formulated and their parameters were optimized so that the resulting models matched as closely as possible many architectural attributes of the corresponding empirical tree models. The hypothesized mechanisms that most closely matched the empirical tree models were then considered more likely to capture the details of real crown development mechanisms. TLS data and QSMs were similarly used by Potapov *et al.* (2016), where they proposed a stochastic version of LIGNUM for producing tree structures consistent with detailed TLS data. They did the matching by iteratively finding the best correspondence between the empirical QSMs and the stochastic choices of the algorithm. They concluded that the proposed approach is a viable solution for realistic plant models based on data and accounting for the stochastic influences. The trees produced with their data-based model resemble the real measured trees and are statistically similar but not copies of each other. Later they expanded the idea to general stochastic structure models and showed how to generate data-based morphological tree structure clones (Potapov *et al.*, 2017).

Figure 6 specifies the principal steps in the TLS to FSPM workflow for simulation of tree communities. Although this paper has shown examples of these steps, there are still many obstacles that have to be overcome in order for the workflow to be an easy one to apply. For example, in step 1, when scanning a forest from multiple positions, there comes a point when the density of woody plants is too great to allow for efficient placement of TLS instruments. This in turn results in high occlusion of woody branches or missing areas entirely if it is not possible to reach all areas of a survey site. One way of overcoming this issue is by using LiDAR-mounted unmanned aerial vehicles (UAVs) to complement ground-based TLS in order to minimize data gaps (Brede *et al*., 2019; Schneider *et al*., 2019). Density of woody plants in a forest stand can also greatly impact step 2. if there is significant branch overlap between neighbouring plants, automated and semi-automated methods may struggle to distinguish which branches belong to which plant. Species can also impact individual isolation from a point cloud if there are many species with a low, shrubby growth habit. Many of the separation algorithms rely on the ability to identify a distinct trunk close to the ground, and if this is not possible manual isolation may be required.

After step 2 the challenge of step 3 and its applications for FSPMs is acquiring botanically accuracy in QSM reconstruction. The way QSMs are reconstructed usually cannot produce a botanically faithful architectural structure. For example, it may be that e.g. internodes are not correctly identified or foliage (if it is detected) is placed in internodes that botanically cannot carry it. For some species and with good enough quality data, it is possible to post-process QSMs to proper botanic form. However, for example if dead branches are broken off the stem, this may not leave any discernible trace in the TLS data and thus it is impossible, at least reliably, to reconstruct botanically correct QSMs. Following this, step 4, deriving architectural metrics, is just one part of FSPM parameterization. In this step, in addition to architectural structure, FSPMs require data for environmental and physiological sub-models. Such data are not available from TLS measurements, except for optical properties of foliage and woody parts. These data can be obtained from other models, flux towers or trait databases or existing literature. Differences between parameter requirements of different of FSPMs will dictate the difficulty of finding these values. In addition, the parameter values vary with growing conditions, e.g. site quality. Therefore, if the TLS scan does not provide information on foliage, the foliage must be generated using additional information contained in parameter values or calculated (Lintunen *et al.*, 2011). If a TLS scan is used to initialize forest structure for a simulation, then also the initial values of all variables in a FSPMs dynamical equations are required (i.e. amount of sapwood). This is best accomplished using a special module of the FSPM that calculates initial values as a function of the architectural structure and parameter values. Lastly, a botanically correct architectural structure, including positions of buds that flush to produce growth (Fig. 2), is preferable for starting of simulation.

## DISCUSSION

TLS has already transformed the way we collect data on forest communities, providing unparalleled information about the spatial arrangement of individuals and their interactions in 3-D space, yet currently largely limited to the detection of static and transient patterns between the diversity of vegetation structure and other community features (Davies and Asner, 2014; D’Urban Jackson *et al.*, 2020). FSPMs are capable of scaling structural relationships between different organs of the same plant to the structural relationships of whole plants in the same community. Using TLS together with FSPMs, in turn, forms a promising toolkit to explore questions involving longer time spans in forest community ecology and evolution. The availability of structural data for a wide range of species, across a wide range of habitats is key for shifting the focus of FSPMs from a practical use to a more paradigmatic one. Indeed, TLS data are already available for forest communities in numerous databases (Guzmán *et al.*, 2020), with more data being collected and openly shared over time. Both TLS and FSPMs are associated with large amounts of data and parameters and high computational demand; however, no other combined method is capable of capturing, testing and investigating the spatiotemporal dynamics of forest communities in such detail. When used in tandem, FSPMs and TLS could be used to test hypotheses for dynamic relationships between biodiversity and productivity, ecosystem functioning and resilience to species losses or invasions in forest communities. Whilst these questions have been explored through several experimental studies, the studies that cover all phases of structural development of existing vegetation have exclusively been carried out with herbaceous plants and so have a limited applicability for forest ecosystems (Naeem *et al.*, 1994; Tilman and Downing, 1994). Several experimental studies of tree diversity have been established but even the oldest ones cover only ~20 years of forest development (https://treedivnet.ugent.be). Time is a difficult barrier to overcome in experimental studies of forest structure and dynamics. Whilst sudden changes in a forest community such as tree fall are easily detectable, long-term patterns may take decades to emerge. Using TLS and FSPMs together to generate models of forest development maintains a high level of structural realism and overcomes the limiting factor of time by simulating changing environmental conditions. The potential of this synergy is exciting but there are still a number of challenges that need to be overcome before it can reach its full potential.

In a recent review of agent-based models in plant science and ecology, Zhang and DeAngelis (2020) state that although FSPMs are set up in such a way that they should be able to address many of the same questions as population-level agent-based models, it is not yet possible to apply FSPMs at the landscape level. It may not be even desirable to use FSPMs at that level. However, scaling up from organ to tree community level is necessary and interesting for problems of forest community ecology and evolution. There are only a few applications of FSPMs that simulate tree communities as interacting individuals and this facet of FSPM research is not yet well developed. However, several of the models in Table 1 are able to cope with multitree simulation. The examples for computing environment requirements in the General features of FSPMs section show that FSPMs are able to simulate stand level forest communities with is no principal impediment to using them in such studies. Available computer resources are not an obstacle to simulating a sizable tree community either.

In order to increase the utility of FSPMs in ecology and evolution of forest communities, the focus of development must shift towards a more general approach. Firstly, physiological processes must be easily parameterized. There are numerous ways of modelling basic physiological processes, and it would be beneficial to be able to parameterize the physiological sub-model depending on the context of the study. The FSPMs of Table 1 have been parameterized for several tree species. The parameterizations are nevertheless very model-specific: using the parameter values in another model requires tedious handwork. If this problem could be alleviated, for example through a parameter database as a part of model website (www.quantitative-plant.org), it would speed up applying FSPMs. Secondly, it is essential to have flexibility with regard to the architectural sub-model. For FSPMs to be broadly applied in the ecology and evolution of woody plants it is imperative that a range of structural forms are easily defined. Of the 21 different architectural forms described by Hallé and Oldemann, only a handful have been featured in published FSPMs (Hallé *et al.*, 1978). However, to exchange architectural sub-models, FSPMs could be better suited to deal with input parameter values. Many models apply a formalism [L-systems (Lindenmayer and Prusinkiewicz, 1990) in the first place] to deal with morphological development. This opens possibilities of automatic transfer and translation of architectural information. Further, there exists a formalism to code architectural information, the multiscale tree graph (MTG, Godin and Caraglio, 1998), that can be used to transfer architectural information between FSPMs (Boudon *et al.*, 2012). Lastly, increasing the use of TLS-derived data is one way of overcoming the lack of structural forms found currently in FSPMs; however, only one published FSPM, HELIOS, features open-source TLS integration software (Bailey, 2019). For FSPMs to be readily scaled up to the population level, a dedicated TLS pipeline must become an integral part of FSPM development.

The sampling design proposed by Wilkes *et al.* (2017) for TLS studies could serve as a reasonable baseline for simulating community-level FSPMs. As interest in using TLS for characterizing forest structure grows, so will the availability of TLS datasets. If TLS could be used to rapidly assess forest plots across a landscape, this could be used to inform multiple FSPM simulations across a larger geographical area. Moreover, repeated scans of the same area can provide invaluable temporal data, important to understanding phenology and sensitive responses over time highlighted by Campos *et al.* (2021). The quality and quantity of structural data provided by TLS give FSPM developers the opportunity to make better decisions about the detail and scale of their models, as they are no longer being constrained by the specific set of parameters available. Data captured by TLS automatically covers several levels of structural detail with nearly the same sampling effort. Thus, the data captured by TLS enable the researcher to flexibly make choices about the level of detail employed for research and also to use the data and models to iteratively check how the ecological or evolutionary predictions depend on the level of detail utilized.

In Fig. 6 we describe a potential TLS to FSPM workflow, starting with a forest point cloud and ending with either architectural input for FSPMs or validation and calibration. At each step of the workflow there are multiple software options available and a crucial step in expanding its uptake is a better understanding of the differences between these software options. Benchmarking and comparison have long been a shortcoming of FSPM development, as noted in several reviews (Louarn and Song, 2020; Zhang and DeAngelis, 2020). Indeed, model complexity, inconsistency between sub-model representation, varied input parameters and a lack of open-source code has hindered a thorough evaluation of models (Table 1). However, a few groups have endeavoured to improve this situation, notably in the area of below-ground FSPM development. Schnepf *et al.* (2020) issued a call for participation for a collaborative effort to compare root FSPMs via a two-step system, firstly by identifying a number of ‘benchmarking scenarios’ and secondly by coupling these scenarios with specific research questions. This incremental method can therefore identify differences arising in step 1 which could impact step 2 in ways that would otherwise go unnoticed. Above-ground FSPMs could greatly benefit from a similar approach, and it will be important in upcoming years to improve communication between groups to tackle this issue.

From a TLS perspective, it is also important to ascertain areas of potential divergence in the workflow such as those related to the instrument used or QSM generation. To date, no formal comparison of TLS instruments exists, and it is possible for different instruments to return different results for the same target (Orwig *et al.*, 2018). Scanning from multiple positions, as suggested in Fig. 4, will reduce bias, but, depending on the terrain and equipment, could significantly increase the scanning time. Furthermore, there are limits to how well TLS instruments can capture fine branches and leaves in real field scenarios where even slight wind could introduce substantial noise. Any errors accumulated during the scanning process will only be exacerbated in downstream automatic and semi-automatic methods (Martin-Ducup *et al.*, 2021). For example, low point densities may produce inaccurate QSMs, and without a manual method of validation it is unclear how this might impact architectural input parameters for FSPMs (Disney *et al.*, 2019). Care must also be taken regarding woody plants that do not have a typical ‘tree-like’ structure. Most of the software available for individual segmentation and QSM construction assume that individuals have a singular trunk. However, forests globally contain a variety of forms and it is important that methods can be applied with equal accuracy across all architectural types. Furthermore, most TLS studies have been conducted in temperate, boreal or tropical forest, with little representation in dry forest or savannahs, where woody plants tend to have a more shrub-like growth habit (Muumbe *et al.*, 2021).

A key difference between using FSPMs in horticulture and agronomy and FSPMs in ecology and evolution is that development will ultimately be driven by the search for emergent patterns. FSPMs have long been designed based on the research question at hand, usually for a specific species or outcome. This has made it relatively straightforward to select appropriate sub-models depending on the context. However, designing FSPMs to represent emergent patterns of forest structure and dynamics will require a thorough evaluation of the sub-models included. Evers *et al.* (2019) suggested guidelines for creating mixed-species FSPMs, noting that any neighbourhood interactions should emerge only as a result of individuals requiring and acquiring resources in a given community, and not as an explicit process in the model. The use of mixed-species FSPMs is a relatively recent direction of research but the empirical observations from TLS studies of vegetation dynamics can hopefully guide model development.

## OUTLOOK AND CONCLUSIONS

Since the emergence of FSPMs over two decades ago, there has been an explosion of research into many different facets of this modelling paradigm, with many successes in agronomy and horticulture. Despite this, applications of FSPMs to forest community ecology and evolution have not proceeded at the same pace. We believe that FSPMs, in combination with TLS-derived data, can finally begin to address ecological and evolutionary dynamics in forests. For the potential of this synergy to be realized, a number of challenges need to be addressed. First, FSPM research already spans many disciplines, including life sciences, computer science and mathematics. However, effectively using FSPMs for forest communities requires coordination between plant ecophysiologists, ecologists and evolutionary biologists. This will be essential for linking detailed organ-level processes with community-level processes to improve the explanatory power of FSPMs. Second, efforts must be made to enhance the generality of FSPMs. TLS-derived data have expanded the structural generality of FSPMs immensely, but general methods of implementing the physiological and environmental sub-models still require attention. One way of improving this is to encourage FSPM developers to produce open-source code and participate in benchmarking efforts. Third, TLS data have largely been collected for ‘tree-like’ plants. For a comprehensive understanding of woody plant form, TLS campaigns need to take place in a variety of forest environments, including tropical dry forests. Both FSPMs and the use of TLS for forest surveying are still in their early days but there is a great deal of potential when these tools are combined. The ability to simulate realistic structure and dynamics in forest communities with FSPMs fulfils a specific niche in the spectrum of vegetation modelling. Phenomena that eventually contribute to regional and ecosystem structural diversity and vegetation patterns ultimately occur on a local scale, and thus it is essential not to neglect the impact of fine-scale changes in forest communities. 

## References

[CIT0001] Åkerblom M , Raumonen P, Mäkipää R, Kaasalainen M. 2017. Automatic tree species recognition with quantitative structure models. Remote Sensing of Environment 191: 1–12.

[CIT0002] Allen MT , Prusinkiewicz P, DeJong TM. 2005. Using L-systems for modeling source-sink interactions, architecture and physiology of growing trees: the L-PEACH model. New Phytologist 166: 869–880. 10.1111/j.1469-8137.2005.01348.x 15869648

[CIT0003] Areces-Berazain F , Hinsinger DD, Strijk JS. 2021. Genome-wide supermatrix analyses of maples (*Acer*, Sapindaceae) reveal recurring inter-continental migration, mass extinction, and rapid lineage divergence. Genomics 113: 681–692. 33508445 10.1016/j.ygeno.2021.01.014

[CIT0004] Asbeck T , Pyttel P, Frey J, Bauhus J. 2019. Predicting abundance and diversity of tree-related microhabitats in Central European montane forests from common forest attributes. Forest Ecology and Management 432: 400–408.

[CIT0005] Auzmendi I , Hanan JS. 2020. Investigating tree and fruit growth through functional–structural modelling: implications of carbon autonomy at different scales. Annals of Botany 126: 775–788. 32433720 10.1093/aob/mcaa098 PMC7489063

[CIT0006] Bailey BN . 2018. A reverse ray-tracing method for modelling the net radiative flux in leaf-resolving plant canopy simulations. Ecological Modelling 368: 233–245.

[CIT0007] Bailey BN . 2019. Helios: a scalable 3D plant and environmental biophysical modeling framework. Frontiers in Plant Science 10: 1185. 31681349 10.3389/fpls.2019.01185 PMC6813926

[CIT0008] Barczi J-F , Rey H, Griffon S, Jourdan C. 2018. DigR: a generic model and its open source simulation software to mimic three-dimensional root-system architecture diversity. Annals of Botany 121: 1089–1104. 29506106 10.1093/aob/mcy018 PMC5906913

[CIT0009] Barker MG , Pinard MA. 2001. Forest canopy research: sampling problems, and some solutions. In: Linsenmair KE, Davis AJ, Fiala B, Speight MR, eds. Forestry sciences. Tropical Forest Canopies: Ecology and Management: Proceedings of ESF Conference, Oxford University, 12–16 December 1998. Dordrecht: Springer Netherlands, 23–38.

[CIT0010] Barmpoutis P , Stathaki T, Lloyd J, Soelma Bessera de Moura M, Fernanda de Sousa Carvalho H. 2018. LiDAR Technology and Linear Dynamical Systems for Classification of Tropical Tree Species. In: Župčić I, Španić N, eds. 29th International Conference on Wood Modification and Technology 2018 Implementation of Wood Science in Woodworking Sector LiDAR. University of Zagreb - Faculty of Forestry, 55–62.

[CIT0011] Barthélémy D , Caraglio Y. 2007. Plant architecture: a dynamic, multilevel and comprehensive approach to plant form, structure and ontogeny. Annals of Botany 99: 375–407. 17218346 10.1093/aob/mcl260 PMC2802949

[CIT0012] Béland M , Widlowski J-L, Fournier RA, Côté J-F, Verstraete MM. 2011. Estimating leaf area distribution in savanna trees from terrestrial LiDAR measurements. Agricultural and Forest Meteorology 151: 1252–1266.

[CIT0013] Beland M , Parker G, Sparrow B, et al. 2019. On promoting the use of lidar systems in forest ecosystem research. Forest Ecology and Management 450: 117484.

[CIT0014] Bentley LP , Stegen JC, Savage VM, et al. 2013. An empirical assessment of tree branching networks and implications for plant allometric scaling models. Ecology Letters 16: 1069–1078. 23800188 10.1111/ele.12127

[CIT0015] Beyer RM , Basler D, Raumonen P, Kaasalainen M, Pretzsch H. 2021. Do trees have constant branch divergence angles? Journal of Theoretical Biology 512: 110567. 33359208 10.1016/j.jtbi.2020.110567

[CIT0016] Bohn FJ , Huth A. 2017. The importance of forest structure to biodiversity–productivity relationships. Royal Society Open Science 4: 160521. 28280550 10.1098/rsos.160521 PMC5319316

[CIT0017] Botkin DB , Janak JF, Wallis JR. 1972. Some ecological consequences of a computer model of forest growth. Journal of Ecology 60: 849.

[CIT0018] Boudon F , Pradal C, Cokelaer T, Prusinkiewicz P, Godin C. 2012. L-Py: an L-system simulation framework for modeling plant architecture development based on a dynamic language. Frontiers in Plant Science 3: 76. 22670147 10.3389/fpls.2012.00076 PMC3362793

[CIT0019] Boudon F , Persello S, Jestin A, et al. 2020. V-Mango: a functional–structural model of mango tree growth, development and fruit production. Annals of Botany 126: 745–763. 32391865 10.1093/aob/mcaa089 PMC7489065

[CIT0020] Boukili VK , Chazdon RL. 2017. Environmental filtering, local site factors and landscape context drive changes in functional trait composition during tropical forest succession. Perspectives in Plant Ecology, Evolution and Systematics 24: 37–47.

[CIT0021] Bouman BAM , van Keulen H, van Laar HH, Rabbinge R. 1996. The ‘School of de Wit’ crop growth simulation models: a pedigree and historical overview. Agricultural Systems 52: 171–198.

[CIT0021a] Brede B , Calders K, Lau A, et al. 2019. Non-destructive tree volume estimation through quantitative structure modelling: Comparing UAV laser scanning with terrestrial LIDAR. Remote Sensing of Environment 233: 111355.

[CIT0022] Brienen RJW , Phillips OL, Feldpausch TR, et al. 2015. Long-term decline of the Amazon carbon sink. Nature 519: 344–348. 25788097 10.1038/nature14283

[CIT0023] Brooker RW , Maestre FT, Callaway RM, et al. 2008. Facilitation in plant communities: the past, the present, and the future. Journal of Ecology 96: 18–34.

[CIT0024] Burt A , Disney M, Calders K. 2018. Extracting individual trees from lidar point clouds using *treeseg*. In: Goslee S, ed. Methods in Ecology and Evolution 10: 438–435.

[CIT0025] Calders K , Newnham G, Burt A, et al. 2015 *a*. Nondestructive estimates of above-ground biomass using terrestrial laser scanning. Methods in Ecology and Evolution 6: 198–208.

[CIT0026] Calders K , Schenkels T, Bartholomeus H, Armston J, Verbesselt J, Herold M. 2015 *b*. Monitoring spring phenology with high temporal resolution terrestrial LiDAR measurements. Agricultural and Forest Meteorology 203: 158–168.

[CIT0027] Calders K , Adams J, Armston J, et al. 2020. Terrestrial laser scanning in forest ecology: expanding the horizon. Remote Sensing of Environment 251: 112102.

[CIT0028] von Caemmerer S , Farquhar GD. 1981. Some relationships between the biochemistry of photosynthesis and the gas exchange of leaves. Planta 153: 376–387. 24276943 10.1007/BF00384257

[CIT0029] Campos MB , Litkey P, Wang Y, et al. 2021. A long-term terrestrial laser scanning measurement station to continuously monitor structural and phenological dynamics of boreal forest canopy. Frontiers in Plant Science 11: 606752. 33488656 10.3389/fpls.2020.606752 PMC7817955

[CIT0030] Canham CD , LePage PT, Coates KD. 2004. A neighborhood analysis of canopy tree competition: effects of shading versus crowding. *Canadian Journal of Forest Research* 34: 778–787.

[CIT0031] Chave J , Andalo C, Brown S, et al. 2005. Tree allometry and improved estimation of carbon stocks and balance in tropical forests. Oecologia 145: 87–99. 15971085 10.1007/s00442-005-0100-x

[CIT0032] Chave J , Réjou-Méchain M, Búrquez A, et al. 2014. Improved allometric models to estimate the aboveground biomass of tropical trees. Global Change Biology 20: 3177–3190. 24817483 10.1111/gcb.12629

[CIT0033] Cieslak M , Lemieux C, Hanan J, et al. 2008. Quasi-Monte Carlo simulation of the light environment of plants. Functional Plant Biology 35: 837–849. 32688836 10.1071/FP08082

[CIT0034] Coelho MTP , Rangel TF. 2018. Neutral community dynamics and the evolution of species interactions. American Naturalist 191: 421–434. 10.1086/696216 29570406

[CIT0035] Collalti A , Ibrom A, Stockmarr A, et al. 2020. Forest production efficiency increases with growth temperature. Nature Communications 11: 5322. 10.1038/s41467-020-19187-w PMC7578801 33087724

[CIT0036] Condés S , Aguirre A, del Río M. 2020. Crown plasticity of five pine species in response to competition along an aridity gradient. Forest Ecology and Management 473: 118302.

[CIT0037] Condit R , Ashton P, Bunyavejchewin S, et al. 2006. The importance of demographic niches to tree diversity. Science 313: 98–101. 16763113 10.1126/science.1124712

[CIT0038] Conn A , Pedmale UV, Chory J, Navlakha S. 2017. High-resolution laser scanning reveals plant architectures that reflect universal network design principles. Cell Systems 5: 53–62. 28750198 10.1016/j.cels.2017.06.017

[CIT0040] Costes E , Smith C, Renton M, et al. 2008. MAppleT: simulation of apple tree development using mixed stochastic and biomechanical models. Functional Plant Biology 35: 936–950. 32688844 10.1071/FP08081

[CIT0041] Côté J-F , Fournier RA, Frazer GW, Olaf Niemann K. 2012. A fine-scale architectural model of trees to enhance LiDAR-derived measurements of forest canopy structure. Agricultural and Forest Meteorology 166–167: 72–85.

[CIT0042] Cournède P-H , Mathieu A, Houllier F, Barthélémy D, de Reffye P. 2008. Computing competition for light in the GREENLAB model of plant growth: a contribution to the study of the effects of density on resource acquisition and architectural development. Annals of Botany 101: 1207–1219. 18037666 10.1093/aob/mcm272 PMC2710279

[CIT0043] Coussement JR , De Swaef T, Lootens P, Steppe K. 2020. Turgor-driven plant growth applied in a soybean functional–structural plant model. Annals of Botany 126: 729–744. 32304206 10.1093/aob/mcaa076 PMC7489068

[CIT0044] Curtis EM , Knight CA, Leigh A. 2019. Intracanopy adjustment of leaf-level thermal tolerance is associated with microclimatic variation across the canopy of a desert tree (*Acacia papyrocarpa*). Oecologia 189: 37–46. 30382387 10.1007/s00442-018-4289-x

[CIT0045] Davies AB , Asner GP. 2014. Advances in animal ecology from 3D-LiDAR ecosystem mapping. Trends in Ecology & Evolution 29: 681–691. 25457158 10.1016/j.tree.2014.10.005

[CIT0046] DeAngelis DL . 2018. Individual-based models and approaches in ecology: populations, communities and ecosystems. UK: Taylor and Francis.

[CIT0047] D’Urban Jackson T , Williams GJ, Walker-Springett G, Davies AJ. 2020. Three-dimensional digital mapping of ecosystems: a new era in spatial ecology. Proceedings of the Royal Society B: Biological Sciences 287: 20192383. 10.1098/rspb.2019.2383 PMC7031661 32075534

[CIT0048] Diamond JS , McLaughlin DL, Slesak RA, Stovall A. 2020. Microtopography is a fundamental organizing structure of vegetation and soil chemistry in black ash wetlands. Biogeosciences 17: 901–915.

[CIT0049] Dias ATC , Rosado BHP, De Bello F, Pistón N, De Mattos EA. 2020. Alternative plant designs: consequences for community assembly and ecosystem functioning. Annals of Botany 125: 391–398. 31678986 10.1093/aob/mcz180 PMC7442394

[CIT0050] Díaz S , Cabido M. 2001. Vive la différence: plant functional diversity matters to ecosystem processes. Trends in Ecology & Evolution 16: 646–655. 10.1016/s0169-5347(01)02181-4 11369096

[CIT0051] Dieleman JA , De Visser PHB, Meinen E, Grit JG, Dueck TA. 2019. Integrating morphological and physiological responses of tomato plants to light quality to the crop level by 3D modeling. Frontiers in Plant Science 10: 839. 31354751 10.3389/fpls.2019.00839 PMC6637845

[CIT0052] Disney M . 2019. Terrestrial LiDAR: a three‐dimensional revolution in how we look at trees. New Phytologist 222: 1736–1741. 10.1111/nph.15517 30295928

[CIT0053] Disney M , Burt A, Calders K, Schaaf C, Stovall A. 2019. Innovations in ground and airborne technologies as reference and for training and validation: terrestrial laser scanning (TLS). Surveys in Geophysics 40: 937–958.

[CIT0054] Disney MI , Boni Vicari M, Burt A, et al. 2018. Weighing trees with lasers: advances, challenges and opportunities. Interface Focus 8: 20170048. 29503726 10.1098/rsfs.2017.0048 PMC5829188

[CIT0055] Douma JC , de Vries J, Poelman EH, Dicke M, Anten NPR, Evers JB. 2019. Ecological significance of light quality in optimizing plant defence: R:FR mediated defence. Plant, Cell & Environment 42: 1065–1077. 10.1111/pce.13524 PMC6392137 30702750

[CIT0056] Du S , Lindenbergh R, Ledoux H, Stoter J, Nan L. 2019. AdTree: accurate, detailed, and automatic modelling of laser-scanned trees. Remote Sensing 11: 2074.

[CIT0057] Dymytrova L , Stofer S, Ginzler C, Breiner FT, Scheidegger C. 2016. Forest-structure data improve distribution models of threatened habitat specialists: implications for conservation of epiphytic lichens in forest landscapes. Biological Conservation 196: 31–38.

[CIT0058] Escudero A , del Río T, Sánchez-Zulueta P, Mediavilla S. 2017. Ontogenetic changes in crown architecture and leaf arrangement: effects on light capture efficiency in three tree species differing in leaf longevity. Ecological Research 32: 595–602.

[CIT0059] Evers JB , van der Werf W, Stomph TJ, Bastiaans L, Anten NPR. 2019. Understanding and optimizing species mixtures using functional–structural plant modelling. Journal of Experimental Botany 70: 2381–2388. 30165416 10.1093/jxb/ery288

[CIT0060] Falster DS , Brännström Å, Westoby M, Dieckmann U. 2017. Multitrait successional forest dynamics enable diverse competitive coexistence. Proceedings of the National Academy of Sciences of the USA 114: E2719–E2728. 28283658 10.1073/pnas.1610206114 PMC5380092

[CIT0061] Farnsworth KD , Niklas KJ. 1995. Theories of optimization, form and function in branching architecture in plants. Functional Ecology 9: 355–363.

[CIT0062] Farquhar GD , von Caemmerer S, Berry JA. 1980. A biochemical model of photosynthetic CO2 assimilation in leaves of C3 species. Planta 149: 78–90. 24306196 10.1007/BF00386231

[CIT0063] Felipe-Lucia MR , Soliveres S, Penone C, et al. 2018. Multiple forest attributes underpin the supply of multiple ecosystem services. Nature Communications 9: 4839. 10.1038/s41467-018-07082-4 PMC6240034 30446752

[CIT0064] Fernández MP , Norero A, Vera JR, Pérez E. 2011. A functional–structural model for radiata pine (*Pinus radiata*) focusing on tree architecture and wood quality. Annals of Botany 108: 1155–1178. 21987452 10.1093/aob/mcr156 PMC3189843

[CIT0065] Fisher R , McDowell N, Purves D, et al. 2010. Assessing uncertainties in a second-generation dynamic vegetation model caused by ecological scale limitations. New Phytologist 187: 666–681. 10.1111/j.1469-8137.2010.03340.x 20618912

[CIT0066] Fisher RA , Koven CD, Anderegg WRL, et al. 2017. Vegetation demographics in Earth System Models: a review of progress and priorities. Global Change Biology 24: 35–54. 28921829 10.1111/gcb.13910

[CIT0067] Francis EJ , Asner GP, Mach KJ, Field CB. 2020. Landscape scale variation in the hydrologic niche of California coast redwood. Ecography 43: 1305–1315.

[CIT0068] Franklin O , Harrison SP, Dewar R, et al. 2020. Organizing principles for vegetation dynamics. Nature Plants 6: 444–453. 32393882 10.1038/s41477-020-0655-x

[CIT0069] Givnish TJ . 1984. Leaf and canopy adaptations in tropical forests. In: Medina E, Mooney HA, Vázquez-Yánes C, eds. Tasks for vegetation science. Physiological ecology of plants of the wet tropics: Proceedings of an International Symposium Held in Oxatepec and Los Tuxtlas, Mexico, June 29 to July 6, 1983. Dordrecht: Springer, 51–84.

[CIT0070] Givnish TJ . 1999. On the causes of gradients in tropical tree diversity. Journal of Ecology 87: 193–210.

[CIT0071] Godin C . 2000. Representing and encoding plant architecture: a review. Annals of Forest Science 57: 413–438.

[CIT0072] Godin C , Caraglio Y. 1998. A multiscale model of plant topological structures. Journal of Theoretical Biology 191: 1–46. 9593655 10.1006/jtbi.1997.0561

[CIT0073] Godin C , Sinoquet H. 2005. Functional–structural plant modelling. New Phytologist 166: 705–708. 10.1111/j.1469-8137.2005.01445.x 15869632

[CIT0074] Grime JP . 2006. Trait convergence and trait divergence in herbaceous plant communities: mechanisms and consequences. Journal of Vegetation Science 17: 255–260.

[CIT0075] Guzmán QJA , Sharp I, Alencastro F, Sánchez‐Azofeifa GA. 2020. On the relationship of fractal geometry and tree–stand metrics on point clouds derived from terrestrial laser scanning. Methods in Ecology and Evolution 11: 1309–1318.

[CIT0076] Hackenberg J , Spiecker H, Calders K, Disney M, Raumonen P. 2015. SimpleTree – an efficient open source tool to build tree models from TLS clouds. Forests 6: 4245–4294.

[CIT0077] Hallé F , Oldeman RAA, Tomlinson PB. 1978. Tropical trees and forests: an architectural analysis. Berlin: Springer.

[CIT0078] Harper JL . 1977. Population biology of plants. London: Academic Press.

[CIT0079] Harper JL . 1980. Plant demography and ecological theory. Oikos 35: 244–253.

[CIT0080] Hemmerling R , Kniemeyer O, Lanwert D, et al. 2008. The rule-based language XL and the modelling environment GroIMP illustrated with simulated tree competition. Functional Plant Biology 35: 739–750. 32688828 10.1071/FP08052

[CIT0081] Henke M , Buck-Sorlin GH. 2017. Using a full spectral raytracer for calculating light microclimate in functional-structural plant modelling. Computing and Informatics 36: 1492–1522.

[CIT0082] Hildebrand M , Perles-Garcia MD, Kunz M, Härdtle W, von Oheimb G, Fichtner A. 2021. Reprint of: tree-tree interactions and crown complementarity: the role of functional diversity and branch traits for canopy packing. Basic and Applied Ecology 55: 53–63.

[CIT0083] Hilmers T , Friess N, Bässler C, et al. 2018. Biodiversity along temperate forest succession. Journal of Applied Ecology 55: 2756–2766.

[CIT0084] Honkaniemi J , Rammer W, Seidl R. 2021. From mycelia to mastodons—a general approach for simulating biotic disturbances in forest ecosystems. Environmental Modelling & Software 138: 104977.

[CIT0085] Host GE , Isebrands JG, Theseira GW, Kiniry JR, Graham RL. 1996. Temporal and spatial scaling from individual trees to plantations: a modeling strategy. Biomass and Bioenergy 11: 233–243.

[CIT0086] Host GE , Stech HW, Lenz KE, et al. 2008. Forest patch modeling: using high performance computing to simulate aboveground interactions among individual trees. Functional Plant Biology 35: 976–987. 32688847 10.1071/FP08075

[CIT0087] Houston A , Clark C, McNamara J, Mangel M. 1988. Dynamic models in behavioural and evolutionary ecology. Nature 332: 29–34.

[CIT0088] Hubbell SP . 2001. A unified theory of biodiversity and biogeography. Princeton: Princeton University Press.

[CIT0089] Ishii HR , Horikawa S, Noguchi Y, Azuma W. 2018. Variation of intra-crown leaf plasticity of *Fagus crenata* across its geographical range in Japan. Forest Ecology and Management 429: 437–448.

[CIT0090] Jackson T , Shenkin A, Kalyan B, et al. 2019. A new architectural perspective on wind damage in a natural forest. Frontiers in Forests and Global Change 1: 13.

[CIT0091] Junttila S , Hölttä T, Puttonen E, et al. 2021. Terrestrial laser scanning intensity captures diurnal variation in leaf water potential. Remote Sensing of Environment 255: 112274.

[CIT0092] Kaitaniemi P , Lintunen A. 2021. Exploring the potential to improve the estimation of boreal tree structural attributes with simple height- and distance-based competition index. Forests 12: 324.

[CIT0093] Kaitaniemi P , Lintunen A, Sievänen R. 2020. Power-law estimation of branch growth. Ecological Modelling 416: 108900.

[CIT0094] Kang H , Fiser M, Shi B, Sheibani F, Hirst P, Benes B. 2016. IMapple—functional structural model of apple trees. In: 2016 IEEE International Conference on Functional-Structural Plant Growth Modeling, Simulation, Visualization and Applications (FSPMA), Quingdao, China, 90–97.

[CIT0095] Kaufmann S , Hauck M, Leuschner C. 2018. Effects of natural forest dynamics on vascular plant, bryophyte, and lichen diversity in primeval *Fagus sylvatica* forests and comparison with production forests. Journal of Ecology 106: 2421–2434.

[CIT0096] Kennedy MC . 2010. Functional–structural models optimize the placement of foliage units for multiple whole-canopy functions. Ecological Research 25: 723–732.

[CIT0097] Kniemeyer O , Kurth W. 2008. The modelling platform GroIMP and the programming language XL. In: Schürr A, Nagl M, Zündorf A, eds. Lecture notes in computer science. Applications of graph transformations with industrial relevance. Berlin: Springer, 570–572.

[CIT0098] Köhler P , Huth A. 1998. The effects of tree species grouping in tropical rainforest modelling: simulations with the individual-based model Formind. Ecological Modelling 109: 301–321.

[CIT0099] Koyama K , Shirakawa H, Kikuzawa K. 2020. Redeployment of shoots into better-lit positions within the crowns of saplings of five species with different growth patterns. Forests 11: 1301.

[CIT0100] Kunz M , Fichtner A, Härdtle W, Raumonen P, Bruelheide H, Oheimb G. 2019. Neighbour species richness and local structural variability modulate aboveground allocation patterns and crown morphology of individual trees. Ecology Letters 22: 2130–2140. 31625279 10.1111/ele.13400

[CIT0101] Lamanna C , Blonder B, Violle C, et al. 2014. Functional trait space and the latitudinal diversity gradient. Proceedings of the National Academy of Sciences of the USA 111: 13745–13750. 25225365 10.1073/pnas.1317722111 PMC4183280

[CIT0102] LaRue EA , Hardiman BS, Elliott JM, Fei S. 2019. Structural diversity as a predictor of ecosystem function. Environmental Research Letters 14: 114011.

[CIT0103] LaRue EA , Wagner FW, Fei S, et al. 2020. Compatibility of aerial and terrestrial LiDAR for quantifying forest structural diversity. Remote Sensing 12: 1407.

[CIT0104] Lau A , Calders K, Bartholomeus H, et al. 2019. Tree biomass equations from terrestrial LiDAR: a case study in Guyana. Forests 10: 527.

[CIT0105] Lehnebach R , Beyer R, Letort V, Heuret P. 2018. The pipe model theory half a century on: a review. Annals of Botany 121: 773–795. 29370362 10.1093/aob/mcx194 PMC5906905

[CIT0106] Lescourret F , Moitrier N, Valsesia P, Génard M. 2011. QualiTree, a virtual fruit tree to study the management of fruit quality. I. Model development. Trees 25: 519–530.

[CIT0107] Letort V , Cournède P-H, Mathieu A, et al. 2008. Parametric identification of a functional–structural tree growth model and application to beech trees (*Fagus sylvatica*). Functional Plant Biology 35: 951–963. 32688845 10.1071/FP08065

[CIT0108] Li W , Guo Q, Tao S, Su Y. 2018. VBRT: a novel voxel-based radiative transfer model for heterogeneous three-dimensional forest scenes. Remote Sensing of Environment 206: 318–335.

[CIT0109] Liang X , Kankare V, Hyyppä J, et al. 2016. Terrestrial laser scanning in forest inventories. ISPRS Journal of Photogrammetry and Remote Sensing 115: 63–77.

[CIT0110] Lindenmayer A , Prusinkiewicz P. 1990. The algorithmic beauty of plants. New York: Springer.

[CIT0111] Lintunen A , Kaitaniemi P. 2010. Responses of crown architecture in *Betula pendula* to competition are dependent on the species of neighbouring trees. Trees 24: 411–424.

[CIT0112] Lintunen A , Sievänen R, Kaitaniemi P, Perttunen J. 2011. Models of 3D crown structure for Scots pine (*Pinus sylvestris*) and silver birch (*Betula pendula*) grown in mixed forest. Canadian Journal of Forest Research 41: 1779–1794.

[CIT0113] Lopez G , Favreau RR, Smith C, et al. 2008. Integrating simulation of architectural development and source–sink behaviour of peach trees by incorporating Markov chains and physiological organ function submodels into L-PEACH. Functional Plant Biology 35: 761–771. 32688830 10.1071/FP08039

[CIT0114] Louarn G , Song Y. 2020. Two decades of functional–structural plant modelling: now addressing fundamental questions in systems biology and predictive ecology. Annals of Botany 126: 501–509. 32725187 10.1093/aob/mcaa143 PMC7489058

[CIT0115] Lu M , Nygren P, Perttunen J, Pallardy S, Larsen D. 2011. Application of the functional-structural tree model LIGNUM to growth simulation of short-rotation eastern cottonwood. Silva Fennica 45: 431–474.

[CIT0116] MacFarlane DW , Kane B. 2017. Neighbour effects on tree architecture: functional trade-offs balancing crown competitiveness with wind resistance. Functional Ecology 31: 1624–1636.

[CIT0117] Mäkelä A , Hari P. 1986. Stand growth model based on carbon uptake and allocation in individual trees. Ecological Modelling 33: 205–229.

[CIT0118] Mäkelä A , Landsberg J, Ek AR, et al. 2000. Process-based models for forest ecosystem management: current state of the art and challenges for practical implementation. Tree Physiology 20: 289–298. 12651445 10.1093/treephys/20.5-6.289

[CIT0119] Mäkelä A , Valentine HT, Helmisaari H-S. 2008. Optimal co-allocation of carbon and nitrogen in a forest stand at steady state. New Phytologist 180: 114–123. 10.1111/j.1469-8137.2008.02558.x 18637066

[CIT0120] Makowski M , Hädrich T, Scheffczyk J, Michels DL, Pirk S, Pałubicki W. 2019. Synthetic silviculture: multi-scale modeling of plant ecosystems. ACM Transactions on Graphics 38: 1–14.

[CIT0121] Malhi Y , Jackson T, Patrick Bentley L, et al. 2018. New perspectives on the ecology of tree structure and tree communities through terrestrial laser scanning. Interface Focus 8: 20170052. 29503728 10.1098/rsfs.2017.0052 PMC5829190

[CIT0122] Marcelis LFM , Heuvelink E, Goudriaan J. 1998. Modelling biomass production and yield of horticultural crops: a review. Scientia Horticulturae 74: 83–111.

[CIT0123] Martin-Ducup O , Schneider R, Fournier RA. 2018. Analyzing the vertical distribution of crown material in mixed stand composed of two temperate tree species. Forests 9: 673.

[CIT0124] Martin-Ducup O , Ploton P, Barbier N, et al. 2020. Terrestrial laser scanning reveals convergence of tree architecture with increasingly dominant crown canopy position. Functional Ecology 34: 2442–2452.

[CIT0125] Martin-Ducup O , Mofack G II, Wang D, et al. 2021. Evaluation of automated pipelines for tree and plot metric estimation from TLS data in tropical forest areas. Annals of Botany 128: 753–765. 10.1093/aob/mcab051 PMC8557371 33876194

[CIT0126] Matkala L , Salemaa M, Bäck J. 2020. Soil total phosphorus and nitrogen explain vegetation community composition in a northern forest ecosystem near a phosphate massif. Biogeosciences 17: 1535–1556.

[CIT0127] May F , Huth A, Wiegand T. 2015. Moving beyond abundance distributions: neutral theory and spatial patterns in a tropical forest. Proceedings of the Royal Society B: Biological Sciences 282: 20141657. 10.1098/rspb.2014.1657 PMC4344136 25631991

[CIT0128] McDowell NG , Allen CD, Anderson-Teixeira K, et al. 2020. Pervasive shifts in forest dynamics in a changing world. Science 368: eaaz9463. 32467364 10.1126/science.aaz9463

[CIT0129] McGill B , Enquist B, Weiher E, Westoby M. 2006. Rebuilding community ecology from functional traits. Trends in Ecology & Evolution 21: 178–185. 16701083 10.1016/j.tree.2006.02.002

[CIT0130] Medlyn B , Barrett D, Landsberg J, Sands P, Clement R. 2003. Conversion of canopy intercepted radiation to photosynthate: review of modelling approaches for regional scales. Functional Plant Biology 30: 153–169. 32689002 10.1071/FP02088

[CIT0131] Mirás-Avalos JM , Egea G, Nicolás E, et al. 2011. QualiTree, a virtual fruit tree to study the management of fruit quality. II. Parameterisation for peach, analysis of growth-related processes and agronomic scenarios. Trees 25: 785–799.

[CIT0132] Mladenoff DJ . 2004. LANDIS and forest landscape models. Ecological Modelling 180: 7–19.

[CIT0133] Moorcroft PR , Hurtt GC, Pacala SW. 2001. A method for scaling vegetation dynamics: the ecosystem demography model. Ecological Monographs 71: 557–586.

[CIT0134] Moorthy SMK , Calders K, Vicari MB, Verbeeck H. 2020. Improved supervised learning-based approach for leaf and wood classification from LiDAR point clouds of forests. IEEE Transactions on Geoscience and Remote Sensing 58: 3057–3070.

[CIT0135] Muumbe TP , Baade J, Singh J, Schmullius C, Thau C. 2021. Terrestrial laser scanning for vegetation analyses with a special focus on savannas. Remote Sensing 13: 507.

[CIT0136] Naeem S , Thompson LJ, Lawler SP, Lawton JH, Woodfin RM. 1994. Declining biodiversity can alter the performance of ecosystems. Nature 368: 734–737.

[CIT0137] Ngao J , Adam B, Saudreau M. 2017. Intra-crown spatial variability of leaf temperature and stomatal conductance enhanced by drought in apple tree as assessed by the RATP model. Agricultural and Forest Meteorology 237–238: 340–354.

[CIT0138] Nikinmaa E , Hölttä T, Hari P, et al. 2013. Assimilate transport in phloem sets conditions for leaf gas exchange. Plant, Cell & Environment 36: 655–669. 10.1111/pce.12004 22934921

[CIT0139] Nock CA , Lecigne B, Taugourdeau O, et al. 2016. Linking ice accretion and crown structure: towards a model of the effect of freezing rain on tree canopies. Annals of Botany 117: 1163–1173. 27107412 10.1093/aob/mcw059 PMC4904176

[CIT0140] Orwig DA , Boucher P, Paynter I, Saenz E, Li Z, Schaaf C. 2018. The potential to characterize ecological data with terrestrial laser scanning in Harvard Forest, MA. Interface Focus 8: 20170044. 29503723 10.1098/rsfs.2017.0044 PMC5829185

[CIT0141] Othmani A , Lew Yan Voon LFC, Stolz C, Piboule A. 2013. Single tree species classification from terrestrial laser scanning data for forest inventory. Pattern Recognition Letters 34: 2144–2150.

[CIT0142] Pacala SW , Deutschman DH. 1995. Details that matter: the spatial distribution of individual trees maintains forest ecosystem function. Oikos 74: 357–365.

[CIT0143] Pacala SW , Canham CD, Silander JA Jr. 2011. Forest models defined by field measurements: I. The design of a northeastern forest simulator. Canadian Journal of Forest Research 23: 1980–1988.

[CIT0144] Pałubicki W , Horel K, Longay S, et al. 2009. Self-organizing tree models for image synthesis. ACM Transactions on Graphics 28: 1–10.

[CIT0145] Pałubicki W , Kokosza A, Burian A. 2019. Formal description of plant morphogenesis. Journal of Experimental Botany 70: 3601–3613. 31290543 10.1093/jxb/erz210

[CIT0146] Peltoniemi MS , Duursma RA, Medlyn BE. 2012. Co-optimal distribution of leaf nitrogen and hydraulic conductance in plant canopies. Tree Physiology 32: 510–519. 22491524 10.1093/treephys/tps023

[CIT0148] Perttunen J . 2001. Application of the functional-structural tree model LIGNUM to sugar maple saplings (*Acer saccharum* Marsh) growing in forest gaps. Annals of Botany 88: 471–481.

[CIT0149] Piboule A , Krebs M, Esclatine L, Hervé JC. 2013. Computree: a collaborative platform for use of terrestrial lidar in dendrometry. In: Proceedings of the International IUFRO Conference MeMoWood, Nancy, France 1–4.

[CIT0150] Picheny V , Casadebaig P, Trépos R, et al. 2017. Using numerical plant models and phenotypic correlation space to design achievable ideotypes. Plant, Cell & Environment 40: 1926–1939. 10.1111/pce.13001 28626887

[CIT0151] Posada JM , Lechowicz MJ, Kitajima K. 2009. Optimal photosynthetic use of light by tropical tree crowns achieved by adjustment of individual leaf angles and nitrogen content. Annals of Botany 103: 795–805. 19151040 10.1093/aob/mcn265 PMC2707872

[CIT0152] Posada JM , Sievänen R, Messier C, Perttunen J, Nikinmaa E, Lechowicz MJ. 2012. Contributions of leaf photosynthetic capacity, leaf angle and self-shading to the maximization of net photosynthesis in *Acer saccharum*: a modelling assessment. Annals of Botany 110: 731–741. 22665700 10.1093/aob/mcs106 PMC3400442

[CIT0153] Potapov I , Järvenpää M, Åkerblom M, Raumonen P, Kaasalainen M. 2016. Data-based stochastic modeling of tree growth and structure formation. Silva Fennica 50: 1–11.

[CIT0154] Potapov I , Järvenpää M, Åkerblom M, Raumonen P, Kaasalainen M. 2017. Bayes forest: a data-intensive generator of morphological tree clones. GigaScience 6: gix079. 10.1093/gigascience/gix079 PMC5632294 29020742

[CIT0155] Pradal C , Dufour-Kowalski S, Boudon F, Fournier C, Godin C. 2008. OpenAlea: a visual programming and component-based software platform for plant modelling. Functional Plant Biology 35: 751. 32688829 10.1071/FP08084

[CIT0156] Prentice IC , Bondeau A, Cramer W, et al. 2007. Dynamic global vegetation modeling: quantifying terrestrial ecosystem responses to large-scale environmental change. In: Canadell JG, Pataki DE, Pitelka LF, eds. Global change—the IGBP series. Terrestrial ecosystems in a changing world. Berlin: Springer, 175–192.

[CIT0157] Prieto JA , Louarn G, Perez Peña J, Ojeda H, Simonneau T, Lebon E. 2020. A functional–structural plant model that simulates whole-canopy gas exchange of grapevine plants (*Vitis vinifera* L.) under different training systems. Annals of Botany 126: 647–660. 31837221 10.1093/aob/mcz203 PMC7489073

[CIT0158] Prusinkiewicz P . 2000. Simulation modeling of plants and plant ecosystems. Communications of the ACM 43: 84–93.

[CIT0159] Prusinkiewicz P . 2004. Art and science of life: designing and growing virtual plants with L-systems. In: Davidson C, Fernandez T. Eds. Nursery Crops: Development, Evaluation, Production and Use: Proceedings of the XXVI International Horticultural Congress. Acta Horticulturae 630, 15–28.

[CIT0160] Raumonen P , 2020. *TreeQSM (v2.4.0)—Reconstruction of quantitative structure models of trees from point cloud data. GitHub*. https://github.com/InverseTampere/TreeQSM.

[CIT0161] Raumonen P , Kaasalainen M, Åkerblom M, et al. 2013. Fast automatic precision tree models from terrestrial laser scanner data. Remote Sensing 5: 491–520.

[CIT0162] de Reffye P , Hu B, Kang M, Letort V, Jaeger M. 2021. Two decades of research with the GreenLab model in agronomy. Annals of Botany 127: 281–295. 32969464 10.1093/aob/mcaa172 PMC7872130

[CIT0163] Richter JP . 1970. The notebooks of Leonardo Da Vinci. USA: Courier Corporation.

[CIT0164] Roussel J-R , Auty D, Coops NC, et al. 2020. lidR: an R package for analysis of airborne laser scanning (ALS) data. Remote Sensing of Environment 251: 112061.

[CIT0165] Le Roux X , Lacointe A, Escobar-Gutiérrez A, Dizès SL. 2001. Carbon-based models of individual tree growth: a critical appraisal. Annals of Forest Science 58: 469–506.

[CIT0166] Ruiz-Benito P , Vacchiano G, Lines ER, et al. 2020. Available and missing data to model impact of climate change on European forests. Ecological Modelling 416: 108870.

[CIT0167] Sarlikioti V , de Visser PHB, Buck-Sorlin GH, Marcelis LFM. 2011. How plant architecture affects light absorption and photosynthesis in tomato: towards an ideotype for plant architecture using a functional–structural plant model. Annals of Botany 108: 1065–1073. 21865217 10.1093/aob/mcr221 PMC3189847

[CIT0168] Scheiter S , Langan L, Higgins SI. 2013. Next-generation dynamic global vegetation models: learning from community ecology. New Phytologist 198: 957–969. 10.1111/nph.12210 23496172

[CIT0168a] Schneider FD , Kükenbrink D, Schaepman ME, Schimel DS, Morsdorf F, 2019. Quantifying 3D structure and occlusion in dense tropical and temperate forests using close-range LiDAR. Agricultural and Forest Meteorology 268: 249–257.

[CIT0169] Schmidt D , Bahr C, Friedel M, Kahlen K. 2019. Modelling approach for predicting the impact of changing temperature conditions on grapevine canopy architectures. Agronomy 9: 426.

[CIT0170] Schnepf A , Leitner D, Landl M, et al. 2018. CRootBox: a structural–functional modelling framework for root systems. Annals of Botany 121: 1033–1053. 29432520 10.1093/aob/mcx221 PMC5906965

[CIT0171] Schnepf A , Black CK, Couvreur V, et al. 2020. Call for participation: collaborative benchmarking of functional-structural root architecture models. The case of root water uptake. Frontiers in Plant Science 11: 316. 32296451 10.3389/fpls.2020.00316 PMC7136536

[CIT0172] Seidel D , Leuschner C, Müller A, Krause B. 2011. Crown plasticity in mixed forests—quantifying asymmetry as a measure of competition using terrestrial laser scanning. Forest Ecology and Management 261: 2123–2132.

[CIT0173] Shinozaki K , Yoda K, Hozumi K, Tatuo K. 1964. A quantitative analysis of plant form – the pipe model theory: I. Basic analysis. Japanese Journal of Ecology 14: 97–105.

[CIT0174] Sievänen R , Nikinmaa E, Nygren P, Ozier-Lafontaine H, Perttunen J, Hakula H. 2000. Components of functional-structural tree models. Annals of Forest Science 57: 399–412.

[CIT0175] Sievänen R , Perttunen J, Nikinmaa E, et al. 2008. Toward extension of a single tree functional–structural model of Scots pine to stand level: effect of the canopy of randomly distributed, identical trees on development of tree structure. Functional Plant Biology 35: 964–975. 32688846 10.1071/FP08077

[CIT0176] Sievänen R , Raumonen P, Perttunen J, Nikinmaa E, Kaitaniemi P. 2018. A study of crown development mechanisms using a shoot-based tree model and segmented terrestrial laser scanning data. Annals of Botany 122: 423–434. 29800102 10.1093/aob/mcy082 PMC6110348

[CIT0177] Da Silva D , Qin L, DeBuse C, DeJong TM. 2014. Measuring and modelling seasonal patterns of carbohydrate storage and mobilization in the trunks and root crowns of peach trees. Annals of Botany 114: 643–652. 24674986 10.1093/aob/mcu033 PMC4156119

[CIT0178] Slavík M , Kuželka K, Modlinger R, Tomášková I, Surový P. 2020. UAV laser scans allow detection of morphological changes in tree canopy. Remote Sensing 12: 3829.

[CIT0179] Smith B , Wårlind D, Arneth A, et al. 2014. Implications of incorporating N cycling and N limitations on primary production in an individual-based dynamic vegetation model. Biogeosciences 11: 2027–2054.

[CIT0180] Song Q , Srinivasan V, Long SP, Zhu X-G. 2020. Decomposition analysis on soybean productivity increase under elevated CO2 using 3-D canopy model reveals synergestic effects of CO2 and light in photosynthesis. Annals of Botany 126: 601–614. 31638642 10.1093/aob/mcz163 PMC7489077

[CIT0181] Sorrensen-Cothern KA , Ford ED, Sprugel DG. 1993. A model of competition incorporating plasticity through modular foliage and crown development. Ecological Monographs 63: 277–304.

[CIT0182] Sterck FJ , Schieving F. 2007. 3-D growth patterns of trees: effects of carbon economy, meristem activity, and selection. Ecological Monographs 77: 405–420.

[CIT0183] Sterck F , Schieving F. 2011. Modelling functional trait acclimation for trees of different height in a forest light gradient: emergent patterns driven by carbon gain maximization. Tree Physiology 31: 1024–1037. 21893522 10.1093/treephys/tpr065

[CIT0184] Sterck FJ , Schieving F, Lemmens A, Pons TL. 2005. Performance of trees in forest canopies: explorations with a bottom-up functional–structural plant growth model. New Phytologist 166: 827–843. 10.1111/j.1469-8137.2005.01342.x 15869645

[CIT0185] Stocker BD , Wang H, Smith NG, et al. 2019. P-model v1.0: an optimality-based light use efficiency model for simulating ecosystem gross primary production. Geoscientific Model Development 13: 1545–1581.

[CIT0186] Svenning J-C . 1999. Microhabitat specialization in a species-rich palm community in Amazonian Ecuador. Journal of Ecology 87: 55–65.

[CIT0187] Terryn L , Calders K, Disney M, et al. 2020. Tree species classification using structural features derived from terrestrial laser scanning. ISPRS Journal of Photogrammetry and Remote Sensing 168: 170–181.

[CIT0188] Tews J , Brose U, Grimm V, et al. 2004. Animal species diversity driven by habitat heterogeneity/diversity: the importance of keystone structures. Journal of Biogeography 31: 79–92.

[CIT0189] Tilman D , Downing JA. 1994. Biodiversity and stability in grasslands. Nature 367: 363–365.

[CIT0190] Trochta J , Krůček M, Vrška T, Král K. 2017. 3D Forest: an application for descriptions of three-dimensional forest structures using terrestrial LiDAR. PLoS ONE 12: e0176871. 28472167 10.1371/journal.pone.0176871 PMC5417521

[CIT0191] Valentine HT , Mäkelä A. 2012. Modeling forest stand dynamics from optimal balances of carbon and nitrogen. New Phytologist 194: 961–971. 10.1111/j.1469-8137.2012.04123.x 22463713

[CIT0192] Ventre-Lespiaucq AB , Escribano-Rocafort AG, Delgado JA, et al. 2016. Field patterns of temporal variations in the light environment within the crowns of a Mediterranean evergreen tree (*Olea europaea*). Trees 30: 995–1009.

[CIT0193] Verbeeck H , Bauters M, Jackson T, Shenkin A, Disney M, Calders K. 2019. Time for a plant structural economics spectrum. Frontiers in Forests and Global Change 2: 43.

[CIT0194] Vermeiren J , Villers SLY, Wittemans L, et al. 2020. Quantifying the importance of a realistic tomato (*Solanum lycopersicum*) leaflet shape for 3-D light modelling. Annals of Botany 126: 661–670. 31840158 10.1093/aob/mcz205 PMC7489060

[CIT0195] Vicari MB, Disney M, Wilkes P, Wilkes A, Wilkes K, Woodgate W . 2019. Leaf and wood classification framework for terrestrial LiDAR point clouds. Methods in Ecology and Evolution 10: 680–694.

[CIT0196] Violle C , Enquist BJ, McGill BJ, et al. 2012. The return of the variance: intraspecific variability in community ecology. Trends in Ecology & Evolution 27: 244–252. 22244797 10.1016/j.tree.2011.11.014

[CIT0197] de Vries J , Poelman EH, Anten N, Evers JB. 2018. Elucidating the interaction between light competition and herbivore feeding patterns using functional–structural plant modelling. Annals of Botany 121: 1019–1031. 29373660 10.1093/aob/mcx212 PMC5906910

[CIT0198] de Vries J , Evers JB, Dicke M, Poelman EH. 2019. Ecological interactions shape the adaptive value of plant defence: herbivore attack versus competition for light. Functional Ecology 33: 129–138. 31007332 10.1111/1365-2435.13234 PMC6472621

[CIT0199] Vos J , Marcelis LFM, Evers JB. 2007. Functional-structural plant modelling in crop production: adding a dimension. Frontis 22: 1–12.

[CIT0200] Vos J , Evers JB, Buck-Sorlin GH, Andrieu B, Chelle M, de Visser PHB. 2010. Functional–structural plant modelling: a new versatile tool in crop science. Journal of Experimental Botany 61: 2101–2115. 19995824 10.1093/jxb/erp345

[CIT0201] Walters RG . 2005. Towards an understanding of photosynthetic acclimation. Journal of Experimental Botany 56: 435–447. 15642715 10.1093/jxb/eri060

[CIT0202] Wang D , Brunner J, Ma Z, et al. 2018. Separating tree photosynthetic and non-photosynthetic components from point cloud data using dynamic segment merging. Forests 9: 252.

[CIT0203] Wang D , Takoudjou SM, Casella E. 2020. LeWoS: a universal leaf-wood classification method to facilitate the 3D modelling of large tropical trees using terrestrial LiDAR. Methods in Ecology and Evolution 11: 376–389.

[CIT0204] Wang F , Letort V, Lu Q, et al. 2012. A functional and structural Mongolian scots pine (*Pinus sylvestris* var. *mongolica*) model integrating architecture, biomass and effects of precipitation. PLoS ONE 7: e43531. 22927982 10.1371/journal.pone.0043531 PMC3425476

[CIT0205] Weiher E , Freund D, Bunton T, Stefanski A, Lee T, Bentivenga S. 2011. Advances, challenges and a developing synthesis of ecological community assembly theory. Philosophical Transactions of the Royal Society B: Biological Sciences 366: 2403–2413. 10.1098/rstb.2011.0056 PMC3130429 21768155

[CIT0206] West GB , Brown JH, Enquist BJ. 1999. A general model for the structure and allometry of plant vascular systems. Nature 400: 664–667.

[CIT0207] West GB , Enquist BJ, Brown JH. 2009. A general quantitative theory of forest structure and dynamics. Proceedings of the National Academy of Sciences of the USA 106: 7040–7045. 19363160 10.1073/pnas.0812294106 PMC2678466

[CIT0208] White J . 1979. The plant as a metapopulation. Annual Review of Ecology and Systematics 10: 109–145.

[CIT0209] Wickman J , Diehl S, Brännström Å. 2019. Evolution of resource specialisation in competitive metacommunities. Ecology Letters 22: 1746–1756. 31389134 10.1111/ele.13338 PMC6852178

[CIT0210] Wilkes P , Lau A, Disney M, et al. 2017. Data acquisition considerations for terrestrial laser scanning of forest plots. Remote Sensing of Environment 196: 140–153.

[CIT0211] Woodgate W , Jones SD, Suarez L, et al. 2015. Understanding the variability in ground-based methods for retrieving canopy openness, gap fraction, and leaf area index in diverse forest systems. Agricultural and Forest Meteorology 205: 83–95.

[CIT0212] Xi Z , Hopkinson C, Rood SB, Peddle DR. 2020. See the forest and the trees: effective machine and deep learning algorithms for wood filtering and tree species classification from terrestrial laser scanning. ISPRS Journal of Photogrammetry and Remote Sensing 168: 1–16.

[CIT0213] Xiao S , Chen S-Y, Wang G. 2007. Does the ESS height of plant population still exist with the inclusion of spatial structure?—An individual-based model research. Ecological Modelling 204: 213–218.

[CIT0214] Van der Zande D , Stuckens J, Verstraeten WW, Muys B, Coppin P. 2010. Assessment of light environment variability in broadleaved forest canopies using terrestrial laser scanning. Remote Sensing 2: 1564–1574.

[CIT0215] van der Zee J , Lau A, Shenkin A. 2021. Understanding crown shyness from a 3-D perspective. Annals of Botany128: 725–735 10.1093/aob/mcab035 PMC8557382 33713413

[CIT0216] Zhang B , DeAngelis DL. 2020. An overview of agent-based models in plant biology and ecology. Annals of Botany 126: 539–557. 32173742 10.1093/aob/mcaa043 PMC7489105

[CIT0217] Zheng G , Moskal LM. 2012. Leaf orientation retrieval from terrestrial laser scanning (TLS) data. IEEE Transactions on Geoscience and Remote Sensing 50: 3970–3979.

[CIT0218] Zhu J , Dai Z, Vivin P, et al. 2018. A 3-D functional–structural grapevine model that couples the dynamics of water transport with leaf gas exchange. Annals of Botany 121: 833–848. 29293870 10.1093/aob/mcx141 PMC5906973

[CIT0219] Zhu X , Skidmore AK, Darvishzadeh R, et al. 2018. Foliar and woody materials discriminated using terrestrial LiDAR in a mixed natural forest. International Journal of Applied Earth Observation and Geoinformation 64: 43–50.

[CIT0220] Zuleta D , Russo SE, Barona A, et al. 2020. Importance of topography for tree species habitat distributions in a terra firme forest in the Colombian Amazon. Plant and Soil 450: 133–149.

